# Supplementary material for: Protective effect of propofol compared with sevoflurane on liver function after hepatectomy with Pringle maneuver: A randomized clinical trial
Source: PLoS One. 2023 Aug 24;18(8):e0290327. doi: 10.1371/journal.pone.0290327 (PMC10449203; doi:10.1371/journal.pone.0290327)
Supplement: S2 Table — ASA-PS: American Society of Anesthesiologists Physical Status, AST: aspartate aminotransaminase, ALT: alanine aminotransaminase, tBil: total bilirubin. Data are presented as the % of the total number or mean [standard deviation], where appropriate. (DOCX) [file pone.0290327.s003.docx]

|  | | | Sevoflurane  n=28 | Propofol  n=28 |
| --- | --- | --- | --- | --- |
| Age (years) | | | 66.3 [12.3] | 64.7 [10.1] |
| Male (%) | | | 67.9 | 71.4 |
| ASA-PS (%) | | 2 | 85.7 | 89.3 |
|  |  | 3 | 14.3 | 10.7 |
| Charlson Comorbidity Index | | | 6.5 [0.7] | 6.6 [0.8] |
| Fatty liver (%) | | | 14.3 | 7.1 |
| Chemotherapy within 1 year (%) | | | 28.6 | 25.0 |
| Primary cancer site (%) | Colorectal cancer | | 75.0 | 78.6 |
|  | Others | | 25.0 | 21.4 |
| Preoperative AST level (IU/l) | | | 27.4 [12.3] | 26.4 [12.3] |
| Preoperative ALT level (IU/l) | | | 24.9 [19.9] | 24.0 [13.7] |
| Preoperative tBil level (mg/dl) | | | 0.8 [0.4] | 0.8 [0.2] |
| Laparoscopic surgery (%) | | | 17.9 | 17.9 |
| The use of epidural anaesthesia (%) | | | 96.4 | 100 |
| Operation time (min) | | | 290.4 [98.2] | 237.4 [46.6] |
| Anaesthesia time (min) | | | 352.0 [106.1] | 305.1 [54.1] |
| Total ischemic time (min) | | | 68.5 [42.7] | 59.7 [27.0] |
| Cycle of Pringle (cycle) | | | 4.4 [3.0] | 3.6 [1.6] |
| Intraoperative bleeding (ml) | | | 607.6 [594.0] | 446.4 [370.7] |
| Intraoperative fluid balance (ml/kg/h) | | | 7.1 [3.3] | 6.8 [2.1] |
| Weight of resected liver (g) | | | 121.5 [97.4] | 158.9 [129.8] |
