# Supplementary material for: Protective effect of propofol compared with sevoflurane on liver function after hepatectomy with Pringle maneuver: A randomized clinical trial
Source: PLoS One. 2023 Aug 24;18(8):e0290327. doi: 10.1371/journal.pone.0290327 (PMC10449203; doi:10.1371/journal.pone.0290327)
Supplement: S3 Table — AST: aspartate aminotransaminase, ALT: alanine aminotransaminase, tBil: total bilirubin, SAE: serious adverse events. Data are presented as the % of the total number or mean [standard deviation], where appropriate. A p-value less than 0.05 was considered to be statistically significant. (DOCX) [file pone.0290327.s004.docx]

|  | Sevoflurane  n=26 | Propofol  n=27 | p value |
| --- | --- | --- | --- |
| Peak AST level (IU/l) | 491.5 [368.0] | 289.4 [153.6] | 0.01 |
| Peak ALT level (IU/l) | 422.4 [266.6] | 263.7 [133.9] | 0.008 |
| Peak tBil level (mg/dl) | 1.3 [0.5] | 1.1 [0.4] | 0.34 |
| SAE (%) | 11.5 | 7.4 | 0.67 |
