# Supplementary material for: Protective effect of propofol compared with sevoflurane on liver function after hepatectomy with Pringle maneuver: A randomized clinical trial
Source: PLoS One. 2023 Aug 24;18(8):e0290327. doi: 10.1371/journal.pone.0290327 (PMC10449203; doi:10.1371/journal.pone.0290327)
Supplement: S4 Table — AST: aspartate aminotransaminase, ALT: alanine aminotransaminase, TIT: total ischemic time, 95%CI: 95% confidence interval. A p-value less than 0.05 was considered to be statistically significant. (DOCX) [file pone.0290327.s005.docx]

| Factor | AST | | | ALT | | |
| --- | --- | --- | --- | --- | --- | --- |
|  | Hazard ratio | 95%CI | P | Hazard ratio | 95%CI | P |
| Operation time | 0.7 | -0.5 to 1.8 | 0.27 | 0.3 | -0.6 to 1.2 | 0.53 |
| TIT | 2.9 | 0.3 to 5.6 | 0.03 | 2.6 | 0.6 to 4.6 | 0.01 |
| Propofol | -150.0 | -294.2 to -5.8 | 0.04 | -126.2 | -234.0 to -18.4 | 0.02 |
