# Supplementary material for: Protective effect of propofol compared with sevoflurane on liver function after hepatectomy with Pringle maneuver: A randomized clinical trial
Source: PLoS One. 2023 Aug 24;18(8):e0290327. doi: 10.1371/journal.pone.0290327 (PMC10449203; doi:10.1371/journal.pone.0290327)
Supplement: S1 File — (DOCX) [file pone.0290327.s007.docx]

**麻酔薬が肝切除術後肝障害に与える影響についてのランダム化比較試験**

**（**Difference between anesthetic agents on liver damages after liver resection**）**

**実施計画書**

**研究代表者　　　：松三　絢弥**

国立がん研究センター中央病院麻酔集中治療科

〒104-0045 東京都中央区築地5-1-1

TEL：03-3542-2511 （内線5822）

FAX：03-3542-2166

E-mail：jmatsumi@ncc.go.jp

**研究事務局　　　：松三　絢弥**

国立がん研究センター中央病院麻酔集中治療科

〒104-0045 東京都中央区築地5-1-1

TEL：03-3542-2511 （内線5822）

FAX：03-3542-2166

E-mail：jmatsumi@ncc.go.jp

作成日・改訂日: 2017年07月01日 計画書案 第1版作成

2017年08月15日 計画書案 第2版作成

2018年01月05日 計画書案 第3版作成

2018年02月01日 計画書案 第4版作成

2018年05月11日 計画書案 第5版作成

2018年05月25日 計画書案 第6版作成

2018年07月10日 計画書案 第7版作成

2018年09月09日 計画書案 第8版作成

2018年10月14日 計画書案 第9版作成

2018年10月17日 計画書案 第9.1版作成

2018年10月31日 計画書案 第9.2版作成

2019年11月 7日 計画書案 第9.3版作成

2020年4月10日 計画書案 第9.4版作成

1. **概要**
   1. **シェーマ**

**転移性肝腫瘍**

5ヶ所以下かつクイノー5区域未満の肝切除術予定

20歳以上90歳未満

術前AST・ALTが100IU/L未満

原発巣切除術・RFA・胆道再建の同時施行なし

**ランダム割付**

**A群：セボフルレン群**

**B群：プロポフォール群**

- 1. **目的**

血行遮断を伴う肝腫瘍切除術後に生じる肝障害に術中使用する麻酔薬が与える影響を明らかにする。

- 1. **対象**

1. 原発巣を問わず、転移性肝腫瘍(疑いを含む)で5ヶ所以下かつ完全切除する肝臓がクイノー5区域未満の肝腫瘍切除術を予定されている。
2. 年齢が20歳以上90歳未満である。
3. 術前のアスパラギン酸アミノトランスフェラーゼ(AST)・アラニンアミノトランスフェラーゼ(ALT)が100 IU/L未満である。
4. 肝切除術と同時に原発巣切除術やラジオ波凝固療法が予定されていない。
5. 肝切除に加えて胆道再建を予定されていない。
6. 試験参加について患者本人から文書で同意が得られている。
   1. **介入**

術前から麻酔導入まで及び麻酔終了以降は両群とも診療上の制限は設けない。また、麻酔中についても麻酔維持として用いる麻酔薬以外に診療上の制限は設けない。

- - 1. **セボフルレン群**

麻酔維持の麻酔薬としてセボフルレンを使用する。使用量は呼気セボフルレン濃度が0.6-2%になるように調整する。

- - 1. **プロポフォール群**

麻酔維持の麻酔薬としてプロポフォールを使用する。使用量は添付文書の用量を遵守して、Bispectral Indexが30-70の範囲になるように調整する。

- 1. **エンドポイント**
     1. **Primary endpoint**

術後3日間測定したASTの最高値。

- - 1. **Secondary endpoint**

術後3日間測定したALTの最高値とJCOG術後合併症基準(Clavien-Dindo分類) v2.0でgradeⅡ以上の術後合併症と術後早期肝機能評価として総ビリルビン値の術後3日以内最高値と設定する。

- 1. **予定登録数と研究期間**

予定登録患者数56人

登録期間：3年3ヶ月、追跡期間：登録終了後1ヶ月、解析期間8ヶ月、総研究期間：4年

- 1. **問い合わせ先**

国立がん研究センター中央病院　麻酔・集中治療科

東京都中央区築地5-1-1

TEL: 03-3542-2511（内線7053）

FAX: 03-3542-2116

担当　松三絢弥

# 目的

目的は、術中使用する麻酔薬が血行遮断を伴う肝腫瘍切除術後に与える影響を明らかにすることである。

# 背景と試験計画の根拠

## 対象

### 背景

肝切除術においてにプリングル法(肝十二指腸靭帯を鉗子などでクランプして肝動脈および門脈系の血流を一時的に遮断する方法)は広く用いられる^1),2)^。プリングル法により術中出血量は減少できるが、肝細胞は虚血再灌流障害(ischemia-reperfusion injury: IRI)を受ける。また、肝切除術のような高侵襲手術では強い酸化ストレスが生じる。IRIや酸化ストレスは術後肝障害の原因となる^3)^。

肝切除術には全身麻酔が必須であるが、吸入麻酔薬を用いる全身麻酔(以下「吸麻」と略)と静脈麻酔薬のみを用いる全身麻酔(total intravenous anesthesia: TIVA)がある。吸入麻酔薬には虚血耐性効果が指摘されており、人工心肺を用いた心臓手術領域において、心筋障害軽減だけでなく術後合併症軽減などの臨床的有用性が認められている^4)^。一方、TIVAで用いられるプロポフォールには抗酸化作用が指摘されているが、^5)^、臨床的有用性は明らかでない。肝臓手術領域において、プロポフォールと比較して吸麻がIRIに対する保護作用がある報告と有意差がないという報告が入り混じっている一方、プロポフォールに抗酸化作用を認めるという報告があり、術後肝障害に関する結果は不均一である^5)-12)^。不均一の原因として、IRIに影響する複数の因子に介入(虚血法と使用麻酔薬の双方に介入)している点、試験対象の不均一さ(背景肝機能の違い、プリングル法の適用方法の違い)が考えられる。

今回、肝切除術において術中使用する麻酔薬が術後肝機能に与える影響についての研究を計画した。肝臓における肝障害の最も良いマーカーはトランスアミナーゼ(AST・ALT)である^13)^。実際に、肝臓領域の臨床研究において肝障害の評価は術後早期のトランスアミナーゼ最高値が用いられている^5)-11)^。しかし、AST・ALTに影響する因子は多い。手術関連因子としては、切除する肝臓の大きさや肝虚血時間に影響される。また、背景肝機能(肝硬変の有無・程度)によって差がある(肝機能低下が強いとIRIが強くなる傾向にある)。したがって、それらの因子のバラツキが比較的小さい、転移性肝腫瘍切除術を選択した。さらに、切除する肝臓の大きさに著明な差が出ないように5ヶ所以下の肝腫瘍切除術を予定されている症例に限定し、さらに完全切除する肝臓がクイノー5区域以上となる場合は除外することする。また、他部位(原発巣など)切除術やラジオ波焼灼療法を同時に行う場合は除外することとした。

本試験で術中使用麻酔薬によって違いがあれば、適切な麻酔薬を選択することで肝切除術後肝障害の軽減に貢献しうる。

### 現在の標準的な全身麻酔法

全身麻酔の方法としては前述のように吸麻とTIVAがある。歴史的には吸麻のみであったが、薬物的・技術的進歩によって十数年前からTIVAを一般的に行うことが可能となった。吸麻で使用される薬剤としてはセボフルランが代表的で、TIVAではプロポフォールが用いられる。TIVAと比較した際の吸麻の主な長所は、①呼気中の濃度を測定することで脳内濃度の推察が可能であるためより確実な鎮静が達成されやすいこと、②麻酔中の薬剤更新頻度が少ないため急性出血など状態変化時の対応が容易であることである。逆に、短所は①悪性高熱症という重篤な病態を生じる可能性がある(発症率1〜2/10万例)こと、②理論的にはがん転移を促進する可能性があることである。吸麻と比較した際のTIVAの主な長所は、①麻酔後の嘔気嘔吐が少ないこと、②麻酔覚醒の質が良好であることである。そして、短所は①大豆・卵と交差反応するアナフィラキシーを生じる可能性があること、②吸麻と比較して薬物に対する反応の個人差が大きく確実な意識状態モニタリング法ないがため鎮静の確実性がやや劣ること、③吸麻と比較してコストが高いことである。

上記のように吸麻とTIVAには一長一短あるため、いずれかの薬剤に既知の禁忌(大豆・卵アレルギー、悪性高熱症の家族歴など)がある場合と人工心肺を必要とする手術(前述のように吸入麻酔薬の臨床的有用性が指摘されている)を除くと、明らかな優劣はないと考えられている。現状では吸麻・ TIVAの選択は麻酔担当医師の任意で行われているため、肝切除術の際の麻酔薬選択の指針作成を意図して本試験を計画した。

## 試験治療設定の根拠

### 本試験の試験治療

上述のように本試験で使用する麻酔薬はいずれも優劣は認められていない。また、過去の試験結果も以下に示すように不均一である。したがって、吸麻とTIVAいずれも標準治療薬と考えられる。

また、本研究の準備段階として行った当院における転移性肝腫瘍症例を対象とした後ろ向き研究では、揮発性麻酔薬の中でセボフルランを使用した症例でトランスアミナーゼが高値(最高AST値：セボフルレン群658±460 vs プロポフォール群 351±178)という結果であった(論文未発表)。

なお、本試験に用いる薬剤は保険診療下に全身麻酔で用いられている、また本試験に用いる検査結果は通常臨床診療で行われたものを用いる。したがって、本試験のために特定の費用は発生しない

| 著者名 | 掲載誌・年 | デザイン | 対象 | 群分け | 結果 | 問題点 |
| --- | --- | --- | --- | --- | --- | --- |
| K. Slankamenacら^10)^ | HPB 2012 | 前向き  観察  研究 | 肝細胞癌切除 | S群141例、  P群86例 | 最高AST(S629±783 vs. P593±696)・ALT・ビリルビン、術後合併症に有意差なし | 麻酔薬選択が麻酔医の任意、  連続虚血  患者背景に違い |
| Beck-Schimnerら^6)^ | Ann Surg 2008 | RCT | 肝細胞癌切除 | P群34例、  P+S虚血前投与群30例 | 最高AST(P+S508±292 vs. P733±637)、術後合併症に有意差(セボフルランに有用性) | 連続虚血、  患者背景に違い |
| Beck-Schimnerら^7)^ | Ann Surg 2012 | RCT | 肝細胞癌切除 | P+連続虚血+S虚血後投与群48例、  P+間欠虚血群50例、  P+連続虚血群17例 | 最高AST(P+連続虚血631[386-741] vs. P+連続虚血+S443[306-644] vs. P+間欠虚血438[303-549])、術後合併症に有意差(プロポフォール+連続肝虚血以外に有用性) | 肝虚血法に違い |
| Beck-Schimnerら^11)^ | Transplantation 2015 | RCT | 死亡肝移植 | レシピエントの麻酔法として  P群48例、S群50例 | 最高AST(P925[512-3274] vs. S1097[540-2633])・ALTに  有意差なし | ドナー介入なし、  連続虚血状態、  虚血時間に違い |
| B. Laviolleら^5)^ | Fundam Clin Pharmac 2012 | RCT | 4区域未満の肝細胞癌切除 | P17例、  D13例 | 抗酸化バイオマーカーに有意差(プロポフォールに有用性)、  最高AST(数値記載なし)・ALTに有意差なし | 肝虚血法に違い |
| Ucar Mら^12)^ | Transplant Proc 2015 | RCT | 生体肝移植  ドナー | P29例、  I 24例 | 抗酸化作用を示すバイオマーカーに有意差(プロポフォールに有用性) | 臨床的検査結果・経過の比較なし |
| JC Songら^8)^ | Anesth and Analg 2010 | RCT | 肝細胞癌切除 | P50例、  S50例 | 最高AST(P581±494 vs. S435±275)・ALT・ビリルビン、術後合併症に有意差なし | 連続肝虚血、  患者背景不明 |

RCT：無作為化試験、P：プロポフォール、S：セボフルレン、D：デスフルレン、I：イソフルレン

### 標準治療と試験治療のリスクバランスとリスク・ベネフィットバランスのまとめ

吸麻とTIVAはともに標準治療である。前述のように、全体的なリスクバランスとリスク・ベネフィットバランスにおいてどちらが優位かは判定できない。

本研究は優越性試験と考えているが、先行研究の結果が不均一であり、どちらの薬剤に優越性が認められるか不明である。

## 試験デザイン

### 計画されている次期試験のデザイン（第I相/第II相試験の場合）

本試験はスクリーニング目的の第II相試験と位置づけられる。先行研究及び当院直近2年間の転移性肝腫瘍切除術を対象とした後ろ向き研究結果からセボフルランとプロポフォールのいずれに優位性が認められるか不明であるため、両側検定を行う。

本試験で麻酔薬により術後肝障害に与える影響が異なるという結果が得られた場合、術後肝障害の原因であるIRIや酸化ストレスは当該臓器だけでなく他臓器を含めた全身に悪影響を与えうるため術後合併症をprimary endpointとした第Ⅲ相試験を計画する。具体的には、麻酔薬の与える影響について、転移性肝腫瘍切除術より重大合併症発症率の高いmajor resectionを対象として術後合併症をprimary endpointとした研究を想定している。

### エンドポイントの設定根拠

### 肝障害の最も良いマーカーはトランスアミナーゼである^13)^。実際に、肝臓領域の臨床研究において肝障害の評価は術後早期のトランスアミナーゼ最高値が用いられている^5)-11)^。当院直近2年間の転移性肝腫瘍切除術を対象とした後ろ向き研究結果から、当院では術後3日以内で最高値となることが確認されたため、術後3日間のトランスアミナーゼ最高値をprimary endpointに設定した。自施設後ろ向き研究を含めた先行研究(特にBeck-Schimnerらの研究結果)からALTと比較してASTの有用性が高いと考え、本試験ではprimary endpointにASTを選択し、ALTは特に重要なsecondary endpointとして解析することとした。麻酔薬によって術後肝障害の程度が異なるという判断基準としては、AST300U/L程度の差を認める場合とした。これは、臨床的に考えて、AST300U/L程度の差があれば麻酔薬選択に大きな影響を与えると考えられるとともに、当院での後ろ向き研究で術後AST最高値はセボフルレンが約600 IU/LでTIVAが約300IU/Lであったため設定した。

IRI・酸化ストレスは全身に悪影響を与えうる病態であり術後合併症は重要な評価項目と考える。しかし、本試験は麻酔薬の影響を評価するために転移性肝腫瘍切除術を対象としたため術後合併症発症率は非常に低いことが予測される(当院直近2年間の転移性肝腫瘍切除術においてJCOG術後合併症基準v2.0 gradeⅡ以上の術後合併症発症率は1%)。したがって、術後合併症はsecondary endpointとした。また、術後肝障害が強いと長期的な肝機能低下を生じる可能性があるため術後総ビリルビン(Bil)最高値は重要なendpointである。しかし、術後重大合併症と同様に長期的な肝機能低下が生じる可能性は非常に低いため、secondary endpointに設定した。なお、術後重大合併症は第Ⅲ相試験を行う場合にはprimary endpointに設定する予定である。

### 臨床的仮説と登録数設定根拠

### 本試験の研究仮説は「血行遮断を伴う転移性肝腫瘍切除術において術中使用麻酔薬によって術後肝障害の程度が異なる」である。この場合、「術中使用麻酔薬間で術後肝障害の程度に違いはない」という帰無仮説の検定を行って棄却されれば、術中使用麻酔薬によって術後肝障害の程度に差があると判断でき、次段階として高度な術後肝障害が生じうるmajor resectionにおいて術後合併症をprimary endpointとした試験施行の蓋然性が得られる。

### 術後ASTは正規分布すると見込んだ。次に、臨床的にAST300I/L程度の差を認めると麻酔法決定に影響すると判断し、後述（「予定登録数・登録期間・追跡期間」参照）する考察に基づいて必要登録数を計算し、両群計56例を予定登録数とし、登録期間3年3ヶ月、追跡期間28日間とした。

### 患者登録見込み

　当院単施設で当該手術症例は年間40例前後で、本試験の肝切除術の条件を満たすものは約7割である。参加同意取得率を6割と見込んで、本試験の年間登録数は17例前後と推測され、予定登録期間は3年3ヶ月の見込みである。

- 1. **試験参加に伴って予想される利益と不利益の要約**
     1. **予想される利益**

本研究に参加する患者自身には特定の利益はないと予想される。

- - 1. **予想される危険と不利益**

本研究に参加する患者が通常診療と異なるのは、全身麻酔の維持麻酔薬として無作為にセボフルレンもしくはプロポフォールに割り振られる点である。しかし、①本研究参加有無に関わらず患者は麻酔使用薬の選択に関して意思決定は行っていない(麻酔担当医が決定している)、②肝切除術を含めた様々な手術でどちらの麻酔法も広く用いられており、当院で行われている手術における全身麻酔として優劣は明らかでない、③通常診療で行った検査結果を使用し、本研究に参加することによる追加の検査及び処置は無い。①〜③の理由により、本研究に参加することによって患者が不利益を被るとは考えられない。

### 本試験の意義

### 本試験で術中使用麻酔薬によって術後肝障害に違いがあれば、適切な麻酔薬を選択することで肝切除術後肝障害の軽減に貢献しうるだけでなく、メディエーターを介した他臓器(肺・腎など)障害の軽減による術後合併症減少効果が期待できる。また、術後肝障害に違いがなければ、他の要素で術中使用麻酔薬を選択することが可能となる。

**3. 本試験で用いる規準・定義**

- 1. **肝切除の種類**
- major resection：クイノー区域分類で3区域以上の肝切除
- minor resection：クイノー区域分類で3区域未満の肝切除
  1. **ASA-PS (American Society of Anesthesiologists physical status)**

アメリカ麻酔学会が定めた術前全身状態分類である。

　Class 1：手術の原因となる疾患以外の合併症がない状態

　Class 2：日常生活活動は正常だが軽度の全身疾患を有する状態

　Class 3：日常生活活動が制限される高度の全身疾患を有する状態

　Class 4：生命を脅かす全身疾患を要する状態

　Class 5：手術をしても救命の可能性が低い瀕死の状態

　Class 6：脳死と診断された状態(脳死臓器移植ドナー)

- 1. **Charlson Index^14)^**

予後予測スコアとして作成されたが、術前合併症について評価するスコアとして広く用いられている^15)^。

| 1点 | 2点 | 3点 | 6点 |
| --- | --- | --- | --- |
| 心筋梗塞 | 片麻痺 | 中等度以上の肝機能障害  (門脈圧亢進症伴う肝硬変) | 転移性  固形がん |
| うっ血性心不全 | 中等度以上の腎機能障害 |  |  |
| 末梢血管疾患 | 糖尿病  (3大合併症あり) |  | 先天性免疫不全症候群 |
| 脳血管疾患 | 固形がん  (過去5年間に転移なし) |  |  |
| 認知症 | 白血病 |  |  |
| 慢性肺疾患 | リンパ腫 |  |  |
| 膠原病 |  |  |  |
| 消化性潰瘍 |  |  |  |
| 軽度肝疾患(軽度の肝硬変,慢性肝炎) |  |  |  |
| 糖尿病  (3大合併症なし) |  |  |  |

- 1. **術後合併症**

JCOG術後合併症規準(Clavien-Dindo分類) gradeⅡ以上と定義する。

# 患者選択規準

以下の適格基準を全て満たし、除外規準のいずれにも該当しない患者を登録適格例とする。

- 1. **適格規準**

1. 原発巣を問わず、転移性肝腫瘍(疑いを含む)で5ヶ所以下かつ完全切除する肝臓がクイノー5区域未満の肝腫瘍切除術を予定されている。
2. 年齢が20歳以上90歳未満である。
3. 術前のアスパラギン酸アミノトランスフェラーゼ(AST)・アラニンアミノトランスフェラーゼ(ALT)が100 IU/L未満である。
4. 肝切除術と同時に原発巣切除術やラジオ波凝固療法が予定されていない。
5. 肝切除に加えて胆道再建を予定されていない。
6. 試験参加について患者本人から文書で同意が得られている。
   1. **除外規準**
7. 使用薬剤に既知の禁忌(大豆・卵アレルギー、悪性高熱症の既往・家族歴など)がある場合

# 登録の手順とランダム化

- 1. **登録の手順**

対象患者が適格規準を全て満たし、除外規準に該当しないことを登録適格性確認票を用いて確認し、手術目的の入院時に登録する。国立がん研究センター中央病院8階麻酔集中治療医局のパスワードで管理されたコンピューターに登録する。

　　患者登録や患者選択規準に関する問い合わせ先

　　研究事務局：松三　絢弥

TEL：03-3542-2511 （内線5822）

FAX：03-3542-2166

- - 1. **登録に関しての注意事項**

1. プロトコール治療開始後の登録は例外なく許容されない。
2. 登録は国立がん研究センター中央病院8階麻酔集中治療科医局内のパスワードで管理されたコンピューターに行う。
3. 適格性は登録適格性確認票を用いて確認し、確認票は国立がん研究センター中央病院8階麻酔集中治療科医局の金庫に保管する(保管期間に関しては「データ保管」の項目を参照)。
4. データが不十分な時は、全て満たされるまで登録は受け付けられない。
5. 登録適格性確認票の内容とコンピューターに登録時データが記入されていることを2名以上の研究者が確認して登録完了とする。
6. データの研究利用の拒否を含む同意撤回があった場合を除いて、一度登録された患者は登録取り消し(データベースから抹消)はなされない。
   1. **ランダム割付と割付調整因子**

登録にあたって群は同意取得及び麻酔に関与しない研究者によってランダムに割り付けられる。

調整因子は設けず、置換ブロック法を用いてランダム割付を行う。

- 1. **登録終了の手続き**

本試験で予定した登録数の達成が見込まれる場合(残り3例未満となった場合)、研究事務局は研究者に周知する。研究者は予定登録数に達した以降は、新規患者への説明は行わない。

研究事務局は、予定登録数が登録された日の翌週の金曜日17時に登録を終了する。

# 治療計画と治療変更規準

## 患者の安全が脅かされない限りにおいて、治療および治療変更は本章の記述に従って行う。

## プロトコールに従えば医学的に危険と判断される場合は担当医の医学的判断に従って治療変更を行う。その場合は、「プロトコール逸脱」となるが、医学的に妥当と判断された場合は「臨床的に妥当な逸脱」とされる。安全性以外の意図で行われた逸脱は「臨床的に妥当な逸脱」とはしない。

## プロトコール治療

麻酔維持中に使用する薬剤は割付薬となるが、その他の使用薬剤の選択と使用量や呼吸循環管理を含めた麻酔管理は麻酔担当医の臨床的判断で行われる。

- プリングル法：肝十二指腸靭帯で肝動脈・門脈血流を15分から30分間遮断し、その後5分間開通させることを1コースとし、必要な回数行う。また、虚血再灌流障害の軽減を目的として全ての患者で初回遮断直前にヒドロコルチゾン100mgを静注する。
- 麻酔導入：鎮静薬としてプロポフォール、筋弛緩薬としてロクロニウム、鎮痛薬としてフェンタニルもしくはレミフェンタニルもしくはその両方を使用する。なおA群の場合は患者入眠後にセボフルランを開始する。
- 麻酔維持：鎮静薬としては割付薬、筋弛緩薬としてロクロニウム、鎮痛薬としてフェンタニル(効果部位濃度を指標に間欠投与)もしくはレミフェンタニルもしくは硬膜外麻酔もしくはそれらの併用を行う。
- 麻酔覚醒：麻酔覚醒時、筋弛緩薬のリバースにスガマデクスを用いる。
- その他：輸液・輸血製剤(アルブミン製剤、赤血球製剤、凝固因子製剤、血小板製剤)使用、人工呼吸器設定など呼吸管理、昇圧薬など循環管理は臨床的必要性に応じて行い、本試験参加による制限は設けない。

### **セボフルラン治療群（A群）**

麻酔導入時のみプロポフォールを使用し、それ以外はセボフルランを用いて鎮静を行う。セボフルランは呼気中濃度0.6-2%を目安に管理する。麻酔中はプロポフォール(麻酔導入時は除く)の使用を禁止するが、他の薬剤に関しては特に制限を設けない。

### **プロポフォール治療群（B群）**

麻酔導入から麻酔終了までプロポフォールを用いて鎮静を行う。プロポフォールの使用量は添付文書の用量を遵守して、BISモニター30-70を目安に調整する。麻酔中はセボフルランの使用は禁止するが、他の薬剤に関しては特に制限を設けない。

## プロトコール治療完了・中止規準

## プロトコール治療完了の定義

　　　　本試験では上記プロトコールから逸脱なく麻酔終了とした場合をプロトコール完了とする。

## プロトコール治療中止の規準

## 各群割り当て薬が被疑薬となる有害事象(アレルギー反応など)を認めた場合にプロトコール治療を中止する。

# 予期される有害事象

- 1. **予期される有害事象**

本試験において予期される有害反応は以下の通り。

- - 1. **薬剤で予期される有害事象**

付表にある薬剤添付文書を参照のこと。

- - 1. **手術により予期される有害反応・手術合併症**

1. 麻酔関連合併症

発熱^*1^、アレルギー反応、気道確保不能、アシドーシス*^1^、高カリウム血症^*1、^せん妄、嗄声、血腫^*2^、カテーテル関連感染^*3^

*1全身麻酔合併症として予期される悪性高熱を想定した項目

*2硬膜外麻酔合併症として予期される硬膜外血腫・脊髄くも膜下血腫を想定した項目

*3硬膜外麻酔合併症として予期される硬膜外膿瘍・脊髄くも膜下膿瘍を想定した項目

1. 手術合併症

他臓器損傷、胆汁瘻(10%程度)、術後出血、感染(10%程度)、肝不全(0.1%未満)、肺塞栓、死亡(0,1%未満)

- 1. **有害事象/有害反応の評価**

有害事象/有害反応の評価には「JCOG術後合併症規準(Clavien-Dindo分類)」を用いる。

- - 1. **有害事象のgrading**

有害事象のgradingに際しては、それぞれGrade 0〜4の定義内容に最も近いものにgradingする。

また、gradeに具体的な処置が記載されている場合は、その臨床的な必要性からgradingする。例えば、患者の胸水が増えており、酸素吸入や胸腔ドレナージが適応となる状況にもかかわらずそれを患者が拒否した場合などがある。こうした場合には、実際に治療が行われたかどうか（what was actually done）ではなく、何がなされるべきであったか（what should be done）という医学的判断に基づいてgradingを行う。

# 評価項目・臨床検査・評価スケジュール

- 1. **登録前収集項目(麻酔科術前外来時の情報を用いる)**

患者背景(年齢、性別、原発がん種、術前合併症[Charlson Index]、術前化学療法の有無[行われた場合は使用薬剤]、アレルギー歴)、ASA-PS、生化学検査(AST・ALT・Bil)

- 1. **観察期間**

観察期間は、両群とも術後合併症評価を行う手術28日後までとする。

いずれの群においてもプロトコール治療が中止された場合、観察期間は設定しない。

- 1. **治療期間中の収集項目**

術式、麻酔法、手術時間、麻酔時間、切除肝個数・総重量、総虚血時間、術中出血量、術中輸液・輸血量、術中尿量

- 1. **治療終了後の評価項目**
     1. **一次評価項目**
- 虚血再灌流障害

術後3日間のASTの最高値で評価する。

なお、通常臨床で行われる術後生化学検査(術後3日間は毎日検査される)の結果を使用し、本研究のための採血・検査は行わない。

- - 1. **二次評価項目**
- 虚血再灌流障害

特に重要な二次評価項目として、術後3日間のALTの最高値を評価する。なお、通常臨床で行われる術後生化学検査(術後3日間は毎日検査される)の結果を使用し、本研究のための採血・検査は行わない。

- 術後早期肝機能評価

術後3日間のBilの最高値で評価する。なお、通常臨床で行われる術後生化学検査(術後3日間は毎日検査される)の結果を使用し、本研究のための採血・検査は行わない。

- 術後合併症

術後28日間のJCOG術後合併症規準(Clavien-Dindo分類) gradeⅡ以上の術後合併症を収集する。

- 1. **評価スケジュール**

|  | 治療前 | 術後 | | | |
| --- | --- | --- | --- | --- | --- |
|  | 麻酔外来時 | 1日目 | 2日目 | 3日目 | 〜 28日 |
| 患者背景 | ○ | - | - | - | - |
| ASA-PS | ○ | - | - | - | - |
| AST | ○ | ○ | ○ | ○ | - |
| ALT | ○ | ○ | ○ | ○ | - |
| Bil | ○ | ○ | ○ | ○ | - |
| 術後合併症 | - | ○ | ○ | ○ | ○ |

1. **効果判定とエンドポイントの定義**

動物実験も含めて、肝臓領域において虚血再灌流障害の評価項目としてはトランスアミナーゼ値が用いられる^13)^。肝臓切除後虚血再潅流障害に関する臨床研究においては術後早期のトランスアミナーゼ最高値が用いられている^5)-11)^。本研究の目的は肝臓領域の虚血再潅流障害の評価であるため、primary endpointとして採用する。自施設後ろ向き研究を含めた先行研究(特にBeck-Schimnerらの研究)からALTと比較してASTの有用性が高いと考え、本試験ではprimary endpointにASTを選択し、ALTは重要なsecondary endpointとして解析することとした。なお、1回の血流遮断時間が15分と30分では術後AST・ALTへの影響に差は認めない^16)^。

IRIは全身に悪影響を与えうる病態であり術後重大合併症は重要な評価項目と考える、しかし、本試験は麻酔薬のIRIに与える影響を評価するために転移性肝腫瘍切除術を対象としたため術後重大合併症発症率は非常に低いことが予測される(当院直近2年間の転移性肝腫瘍切除術術後重大合併症発症率は1%)。したがって、secondary endpointとした。また、虚血再灌流障害障害の程度が強いと術後早期から肝障害を生じる可能性があるためBilも重要なendpointである。しかし、術後重大合併症と同様に術後早期肝障害が生じる可能性は非常に低いため、secondary endpointに設定した。なお、術後合併症は第Ⅲ相試験を行う場合にはprimary endpointに設定する予定である。

- 1. **解析対象集団の定義**

解析対象はintention to treatの原則に従い、無作為化後された全例からなる集団から、ランダム化後のprimary endpointに関するデータが全くない症例を除いた集団(最大の解析対象集団[Full Analysis Set: FAS])とする。

また、プロトコール治療中止が発生した場合は、プロトコール治療完了した症例からなる集団(Per Protocol Set: PPS)での解析も行う。

- 1. **エンドポイントの定義**
- Primary endpoint

術後3日以内のAST最高値とする。

当院直近2年間の転移性肝腫瘍切除術を対象とした後ろ向き研究で術後3日以内に術後早期の最高値が達成されることを確認したため、術後3日以内の最高値primary endpointとして設定した。

- Secondary endpoint

重要なsecondary endpointとして、術後3日以内のALT最高値を設定する。

それ以外のendpointとして、JCOG術後合併症規準(Clavien-Dindo分類) GradeⅡ以上の術後合併症と肝機能評価としての術後3日以内のBil最高値を用いる。

1. **有害事象および不具合の報告**

「重篤な有害事象」または「予期されない有害事象」が生じた場合、担当医は研究事務局/研究代表者へ報告する。なお、添付文書にある事象は本研究においては有害事象としてカウントしない

なお、「医薬品、医療機器等の品質、有効性及び安全性の確保等に関する法律」に基づく副作用などの厚生労働大臣への報告」（宛先：医薬品医療機器総合機構安全第一部安全性情報課FAX:0120-395-390、Email: anzensei-hokoku@pmda.go.jp）^17)^、「人を対象とする医学系研究に関する倫理指針」(平成29年文部科学省・厚生労働省告示第1号)^18)^に基づく重篤な有害事象などの各施設の医療機関の長への報告、予期されない重篤な有害事象の医療機関の長から厚生労働大臣等への報告、医療機関から企業への副作用に関する連絡については、研究代表者の責任において適切に行う。

- 1. **緊急報告義務のある有害事象**

以下のいずれかに該当する有害事象を「重篤」とする。

1. プロトコール治療中の死亡で、プロトコール治療との因果関係の有無は問わないあらゆる死亡。
2. プロトコール治療終了後の死亡で、プロトコールとの因果関係が否定できない死亡。明らかな原病死は該当しない。
3. 生命を脅かすもの

CTCAE v4.0におけるGrade4の非血液毒性あるいはこれに相当するものとする。

治療のための入院または入院期間の延長が必要とされる場合。

ただし、以下の目的で入院した場合は重篤な有害事象とは取り扱わないこととする。

- - - 遠隔地から受診する患者の負担を軽減する目的の入院
- 事前に計画された入院

1. 永続的または顕著な障害・機能不全に陥るもの。
2. 再生不良性貧血。骨髄異形成症候群、二次がん等、後世代における先天性の疾病または異常。
3. その他重大な医学的事象。

上記の1)〜6)のいずれにも該当しないが、医学的に重要と研究責任/分担医師が判断するもの。

- 1. **研究代表者の報告義務と報告手順**
     1. **緊急報告**

緊急報告の対象となる有害事象が発生した場合は、担当医は速やかに研究事務局/研究代表者に伝える。

その際、診療録番号等が含まれないよう留意する。

1. CTCAE v4.0-JCOGあるいはClavien-Dindo分類Grade3以上の有害事象、または、その他の医学的に重要な状態と判断される有害事象の発生を知った担当医は速やかに研究事務局/研究代表者に報告する。
2. 追加報告

上記の報告を行った後に新たな情報が得られた場合は、所定の様式に情報を追記し随時報告する。

- - 1. **医療機関の長に対する報告**

緊急報告の対象となる有害事象が発生した場合、研究代表者は「人を対象とする医学系研究に関する倫理指針」における「重篤な有害事象」として、医療機関の規定に従い医療機関の長に対し報告する。

- - 1. **その他の報告先に対する報告**

医薬品・医療機器・再生医療等製品安全生情報の報告：医薬品、医療機器等の品質、有効性及び安全生の確保等に関する法律第68条の10第2項に基づき、報告の必要があると判断した情報については、医療機関の規定に従って適切に厚生労働大臣に報告を行う。

- 1. **研究代表者/研究事務局の責務**
     1. **登録停止と緊急通知の必要性の有無の判断**

研究代表者から報告を受けた研究事務局は、報告内容の緊急性、重要性、影響の程度等を判断し、必要に応じて登録の一時停止や施設内への周知事項の緊急通知の必要性の有無の判断を病院長及び理事長へ委ねる。

- - 1. **施設内の研究者への通知**

研究事務局/研究代表者は、重篤な有害事象について施設内の研究者に研究事務局/研究代表者の判断を文書(電子メール可)にて通知する。

1. **統計的事項**
   1. **主たる解析と判断規準**

主たる解析はA群とB群の術後3日以内のAST最高値をt検定で2群比較し、有意差0.05未満を統計学的有意と判断する。麻酔薬によってIRIの程度が異なるという判断基準としては、AST300U/L程度の差を認める場合とした。これは、AST300U/L程度の差があれば臨床的な麻酔薬選択に大きな影響を与えると考え設定した。

必要に応じて、感度解析として、プロトコール治療を完了した症例からなる集団(PPS)を対象とした解析、あるいは無作為化された全例からなる集団を対象としprimary endpointのデータが欠測となっている症例においてはA群・B群全体で観察された最悪値で補完した解析を行う。

- 1. **予定登録数・登録期間・追跡期間**

現在IRIを最も軽減する虚血方法は間欠虚血法と考えられており、IRIの全身に与える影響を考慮して当院でも間欠虚血法が採用されている。しかし、先行研究は2012年のBeck-Schimnerらの試験の1群を除いてIRIが強く発現する連続虚血を用いており、術後AST値の平均値・中央値・標準偏差・四分位値は間欠虚血後となる本試験の結果とは異なる可能性が高い。したがって、先行研究で唯一の間欠虚血後の結果と当院後ろ向き研究の結果を主に参考し、それ以外の研究は副次的な参考にとどめた。まず、術後ASTは正規分布として扱った。次に、臨床的に考えてAST300U/L程度の差を認めると麻酔薬決定に大きな影響を与えると判断した。また、ASTの標準偏差は300U/Lと見込んだ。最後に、有意差0.05、検知力0.8に設定した。この条件で、各群28例(総計56例)が必要と算出された。

当院単施設で当該手術症例は年間40例前後で、うち5ヶ所以下かつ完全切除する肝臓がクイノー5区域未満の肝腫瘍切除術は約7割である。参加同意取得率を6割と見込んで、本試験の年間登録数は17例前後と推測され、予定登録期間は3年3ヶ月の見込みである。追跡期間は術後合併症の定義である術後28日間とする。

- 1. **中間解析と試験の早期中止**

　　　　　中間解析および試験の早期中止は予定しない。

- 1. **SECONDARY ENDPOINTS の解析**

重要なsecondary endpointとして虚血再灌流障害に関して術後3日以内のALT最高値をt検定で2群比較し、有意差0.05未満を統計学的有意とする。

28日以内の術後合併症に関して、Fisher検定で2群比較し、有意差0.05未満を統計学的有意とする。

肝機能に関して、術後3日以内のビリルビン最高値をt検定で2群比較し、有意差0.05未満を統計学的有意とする。

- 1. **最終解析**

10.1.および10.4.に記載した解析に加え、麻酔法以外がIRIに与える影響について除外するために、患者背景・手術関連項目の比較のうちでp=0.1未満であった項目で多変量ロジスティック分析を行う。

なお、最高トランスアミナーゼ値が正規分布しない可能性は否定できないため、副次的解析としてASTとALTについてWilcoxon検定も行う。

- 1. **研究終了**

　本試験に関連したあらゆる論文の公表をもって研究終了とする。

# 倫理的事項

- 1. **患者の保護**

本研究は人を対象とする医学系研究に関する倫理指針(2016年)及びヘルシンキ宣言(2013年改訂)及び個人情報保護法(2017年)を遵守して実施する。

- 1. **インフォームドコンセント**
     1. **患者への説明**

患者登録に先立って、研究者は研究倫理審査委員会で承認が得られた説明文書(別紙1)を患者本人に渡し、以下の内容を口頭で詳しく説明する。

1. 臨床試験と、説明文書について

本研究が臨床試験であること

1. 参加の自由について

研究参加に先立っての同意拒否が自由であること、いったん同意した後の撤回も自由であり、それらにより不当な診療上の不利益を受けないこと。

1. この臨床試験の対象となる方の病状と治療について

転移性肝腫瘍もしくは疑いで肝切除を予定された方を対象とすること。

1. この臨床試験の意義と目的について
2. この臨床試験の方法

全身麻酔で持続的に使用する麻酔薬を無作為に選択すること。麻酔薬の違いを除いては通常診療と変わらないこと。検査結果や治療内容など医学的情報について収集すること。

1. 予想される利益と可能性のある不利益について

試験に参加することによって享受できると思われる利益と被る可能性のある不利益に関する説明。

1. 臨床試験全体の実施予定期間とあなたに参加いただく期間

参加期間が手術中から28日後までであること。

1. あなたが負担する費用について

一般診療と同様であることの説明：治療にかかる費用は保険制度でまかなわれること。

1. 健康被害が発生した場合の対応・保証について。

健康被害が生じた場合の補償は一般診療での対処に準ずること。

1. 個人情報の取り扱いについて

個人情報の保護に最大の注意が払われること。

1. 研究結果の公表について

個人を特定できない形で学術誌・学会で公表すること。研究の概要・進捗状況・主な結果をUMINで公開すること。ご本人からの情報公開に支障ない範囲で応じること。

1. この臨床試験の資金と利益相反について

本研究は国立がん研究センター研究開発費を資金源とすること。当施設および本研究に関与する研究者は、本研究に関連する企業や団体などと研究の信頼性を損ねるような利益相反を有していないこと。また、本研究によって特定の個人が収益を得ることはないこと。

1. この臨床試験の倫理審査について

国立がん研究センター研究倫理審査委員会の承認を得ていること

1. 研究組織・連絡先

本研究の実施主体は国立がん研究センター中央病院麻酔集中治療科であり、説明・同意取得者は麻酔集中治療科所属の医師となること。

1. 当院での連絡先（相談窓口）
   - 1. **同意**

研究について説明を行い、患者が研究の内容をよく理解したことを確認した上で、研究への参加について依頼する。患者本人が研究参加に同意した場合、同意書(別紙1)に患者本人による署名を得る。研究者は同意書に、説明を行った研究者名と説明日、説明を受け同意した患者名、同意日の記載があることを確認する。

同意書は1部コピーし、コピーを患者本人に手渡し、原本は研究代表者が麻酔集中治療科医局内のパスワードにより施錠された金庫にて厳重に保管する。

- 1. **個人情報の保護と患者識別**
     1. **本研究が従うポリシー・法令・規範**

本研究を行うにあたり、以下の法令・規範に従う。下記以外の法令・規範・ポリシーが適応となる場合は加えて従うこととする。

1. 個人情報の保護に関する法律(平成15年5月30日法律第57号、最終改正:平成27年9月9日法律第65号)
2. 人を対象とする医学系研究に関する倫理指針(平成26年文部科学省・厚生労働省告示第3号)
3. ヘルシンキ宣言(日本医師会訳)
   - 1. **個人情報の利用目的と利用する項目、および利用方法**
4. **利用目的**

本研究では、臨床研究の正しい結果を得るために患者個人を特定して調査を行う目的、および取得した情報を適切に管理する目的で、患者の個人情報を利用する。

1. **利用する項目**

患者の同定・照会のために必要最低限な項目(カルテ番号と診療情報)とする。それ以外の個人情報が収集されることはなく、誤って収集された場合には破棄するかマスキングなど判読不能とする適切な処理を行った上で保管する。

1. **利用方法**

患者の個人情報および診療情報は研究者がデータベースに記入することで収集する。データベースには患者の同定・照会のため収集するカルテ番号と紐づけられた研究登録番号を使用し、対応表は国立がん研究センターが定める規定（「人を対象とした医学系研究の情報の保管に関する標準業務手順書」）に従って、研究責任者もしくは研究責任者が指名する担当者が保管する。また、データベースはパスワードにより制御化された単一のコンピューターを使用する(データを複数のコンピューターで扱わない)。

- 1. **データ保管**

診療録及び診療所記録(電子カルテデータ)は国立がん研究センターが定める規定(「中央病院診療記録管理規定」)に従って保管する。「当該情報に係る資料(症例報告書、研究対象者が作成する記録、修正記録など)」、「その他の資料(研究機関の長による研究申請や研究計画改訂などの結果通知書、実施状況報告書、有害事象報告書、研究計画書や説明同意文書の各版など)」は国立がん研究センターが定める規定(「人を対象とした医学系研究の情報の補完に関する標準業務手順書」)に従って、本研究に関するデータの保管期間は研究終了から10年とし、期限を過ぎた後も出来るだけ長期に保管する。保管機関経過後、本研究に関する試料及び情報を破棄する場合は、匿名化したのち廃棄する。

Primary endpointやsecondary endpointの解析結果に関する論文公表を行った場合には論文PDFを国立がん研究センター中央病院研究企画推進部企画管理室に提出する。

- - 1. **データの二次利用について**

本研究で得られたデータについては、個人識別情報とリンクしない形でデータを二次利用することがあり得る。

- - 1. **安全管理責任体制**

プライバシー保護管理責任者およびプライバシー保護担当者を定め、個人情報の利用にあたっては情報の流出リスクを最小化すべく各種安全管理対策を講じる。

- - 1. **患者情報の開示等に対する対応**

患者本人より本研究組織が有するプライバシーに関する情報の開示などを求められた場合の対応者は、原則として担当者とする。

- - 1. **一般的な問い合わせおよび苦情の受付**

一般的な問い合わせや苦情は、下記にて郵便・電子メール・電話のいずれかの方法で受け付ける。

問い合わせ窓口：国立がん研究センター中央病院　麻酔集中治療科　松三　絢弥

郵送先：〒104-0045 東京都中央区築地5-1-1

E-mail: jmatsumi@ncc.go.jp

電話：03-3542-2511（代）　（担当：松三）

対応時間：月～金　午前8時30分から午後5時15分

- 1. **プロトコールの遵守**

本研究に参加する研究者は、患者の安全と人権を損なわない限り、本プロトコールを遵守する。

- 1. **倫理審査委員会の承認**

本研究の開始に先立ち、研究代表者は、実施計画書、同意説明文書などの文書を国立がん研究センター研究倫理審査委員会の承認と理事長の研究許可を受ける。また、実施計画書、同意説明文書などの文書を改定する場合には、本研究を継続して行うことの適否について、研究倫理審査委員会の承認を得なければならない。

- 1. **臨床研究の進捗状況等報告**

本臨床研究の進捗状況ならびに有害事象などの発生状況を1年に１回、病院長に報告する。

- 1. **プロトコールの内容変更について**

実施計画書や説明文書の変更・改訂を行う場合は，変更内容の発効に先立って研究倫理審査委員会の承認を得る。

- 1. **研究に関わる利益相反(COI)について**

当施設および本研究に関与する研究者の利益相反はがん研究センター利益相反委員会事務局が管理している。当施設および本研究に関与する研究者は、本研究に関連する企業や団体などと研究の信頼性を損ねるような利益相反を有していない。また、本研究によって特定の個人が収益を得ることはない。

- 1. **補償について**

本研究の参加中または終了後に、本研究に参加したことが原因となって予測しなかった健康被害が生じた場合には、通常の診療と同様に適切に対処する。その際の医療費は患者が加入する保険診療内で行うものとし、本研究における補償金は発生しない。

本研究に参加することで生じた健康被害については、通常の診療と同様に病状に応じた適切な治療を保険診療として提供する。その際、医療費の自己負担分については患者の負担とする。また、見舞金や各種手当てなどの経済的な補償は行わない。

- 1. **知的財産について**

本臨床研究により得られた結果やデータ、知的財産権は国立がん研究センターに帰属する。

- 1. **本試験に関する情報公開**

患者本人より本研究組織が有するプライバシーに関する情報の開示などを求められた場合の対応者は、原則として研究者とする。患者本人の希望があれば、本研究全体の成果のフィードバックを行うが、論文掲載の内容となることを説明する。研究成果のフィードバックおよび公表の際、研究対象者が特定されることは一切ないものとする。

また、本試験の概要、進捗状況、主な結果はUMIN-CTR(www.umin.ac.jp/ctr/)で公開する。

# モニタリングと監査

- 1. **定期モニタリング**

研究者責任者が指定した麻酔・集中治療科医師による年1回のモニタリングを行い、国立がん研究センター中央病院モニタリングレポートテンプレートに従って報告する。

また、国立がん研究センター臨床研究支援部門研究企画推進部企画管理室のモニタリング担当者によって、研究実施許可日から2ヶ月後および予定登録期間の半分を満たした時点の2回、直接カルテや各種文書を閲覧するサイトモニタリングを実施する

- 1. **監査**

国立がん研究センター研究監査室が実施する内部監査を受け入れる。

# 未承認薬・未承認医療機器、適応外医薬品・適応外医療機器、保険診療で認められていない医療行為の有無について

未承認薬・医療機器、適応外医薬品・適応外医療機器、保険診療で認められていない医療行為は含まれない。

# 特記事項

なし

# 研究組織

- 1. **研究代表者**

国立がん研究センター中央病院　麻酔集中治療科　松三　絢弥

- 1. **研究事務局**

国立がん研究センター中央病院　麻酔集中治療科

東京都中央区築地5-1-1

TEL: 03-3542-2511（内線7053）

FAX: 03-3542-2116

担当　松三　絢弥

- 1. **研究施設**

国立がん研究センター中央病院

- 1. **本研究の資金源**

国立がん研究センター研究開発費(29-A-12、高齢がん患者の周術期管理とPatient Flow Management最適化の研究、国立がん研究センター中央病院麻酔・集中治療科　佐藤哲文、平成29年度)を資金源とする。

- 1. **プロトコール作成**

国立がん研究センター中央病院　麻酔集中治療科　松三 絢弥

国立がん研究センター中央病院　麻酔集中治療科　佐藤 哲文

国立がん研究センター中央病院　肝胆膵外科　島田 和明

国立がん研究センター中央病院　肝胆膵外科　江﨑 稔

国立がん研究センター中央病院　肝胆膵外科　奈良 聡

国立がん研究センター中央病院　肝胆膵外科　岸 庸二

- 1. **研究者**

国立がん研究センター中央病院　麻酔集中治療科　佐藤 哲文

国立がん研究センター中央病院　麻酔集中治療科　松三 絢弥

国立がん研究センター中央病院　肝胆膵外科　島田 和明

国立がん研究センター中央病院　肝胆膵外科　江﨑 稔

国立がん研究センター中央病院　肝胆膵外科　奈良 聡

国立がん研究センター中央病院　肝胆膵外科　高本 健史

# 研究結果の発表

本試験に関連したあらゆる論文の公表後、結果を速やかに研究機関の長へ報告する。なお、本研究の結果は、国内外の学会およびレフリーのある医学誌で論文にて発表する予定である。原則として、研究結果の主たる公表論文の著者は筆頭を研究代表者とし、それ以下は論文の投稿規定による制限に従って、貢献度に応じでグループ代表者が決定し、最終著者は佐藤哲文とする。

# 参考文献

1. Pringle JHV. Notes on the arrest of hepatic hemorrhage due to trauma. Ann Surg. 1908: 48; 541-549
2. van der Bilt JD, et al. European survey on the application of vascular clamping in liver surgery. Dig Surg. 2007; 24: 423-435.
3. Clavien PA, et al. Strategies for safer liver surgery and partial liver transplantation. N Engl J Med. 2007; 356: 1545-1559
4. Uhlig C, et al. Effects of volatile anesthetics on mortality and postoperative pulmonary and other complications in patients undergoing surgery. Anesthesiology 2016; 124: 11230-1245.
5. Laviolle B, et al. Effect of an anesthesia with propofol compared with desflurane on free radical production and liver function after partial hepatectomy. Fundam Clin Phrmacol 2012; 26: 735-742.
6. Beck-Schimmer B, et al. A randomized controlled trial on pharmacological preconditioning in liver surgery using a volatile anesthetic. Ann Surg 2008; 248: 909-918.
7. Beck-Schimmer B, et al. Protection of pharmacological postconditioning in liver surgery. Ann Surg 2012; 256: 837-845.
8. Song JC, et al. A comparison of liver function after hepatectomy with inflow occlusion between sevoflurane and propofol anesthesia. Anesth Analg 2010; 111: 1036-1041.
9. Ko JS, et al. The effects of desflurane and propofol-remifentanil on postoperative hepatic and renal functions after right hepatectomy in liver donors. Liver transpl 2008; 14: 1150-1158.
10. Slankamenac K, et al. Does pharmacological conditioning with the volatile anaesthetic sevoflurane offer protection in liver surgery? HPB 2012; 14: 854-862.
11. Beck-Schimmer B, et al. Conditioning With Sevoflurane in Liver Transplantation: Results of a Multicenter Randomized Controlled Trial. Transplantation 2015; 99: 1606-1602.
12. Ucar M, et al. Comparison of antioxidant effects of isoflurane and propofol in patients undergoing donor hepatectomy. Transplant Proc 2015; 47: 469-472
13. Iu S, et al. Markers of allograft viability in the rat: relationship between transplantation viability and liver function in the isolated perfused rat liver. Transplantation 1987; 45: 562-569.
14. Charlson ME, et al. A new method of classifying prognostic comorbidity in longitudinal studies: development and validation. J Chronic Dis. 1987; 40: 373-383.
15. Khandoga A, et al. Differential significance of early surgical complications for acute and long-term recurrence-free survival following surgical resection of hepatocellular carcinoma: do comorbidities play a role? Eur J Gastroenterol Hepatol. 2017; 29: 1045-1053.
16. Esaki M, et al. Randomized clinical trial of hepatectomy using intermittent pedicle occlusion with ischaemic intervals of 15 versus 30 minutes. Br J Surg. 2006; 93: 944-951.
17. 「医薬品、医療機器等の品質、有効性及び安全性の確保等に関する法律」に基づく副作用などの厚生労働大臣への報告」 http://www.info.pmda.go.jp/info/houkoku.html
18. 「人を対象とする医学系研究に関する倫理指針」http://www.mhlw.go.jp/stf/seisakunitsuite/bunya/hokabunya/kenkyujigyou/i-kenkyu/index.html
