# Supplementary material for: Protective effect of propofol compared with sevoflurane on liver function after hepatectomy with Pringle maneuver: A randomized clinical trial
Source: PLoS One. 2023 Aug 24;18(8):e0290327. doi: 10.1371/journal.pone.0290327 (PMC10449203; doi:10.1371/journal.pone.0290327)
Supplement: S2 File — (DOCX) [file pone.0290327.s008.docx]

**Protocol**

Difference between anesthetic agents on liver damages after liver resection

**Principal investigator： Junya Matsumi**

National Cancer Center Hospital

Department of Anesthesia and Intensive Care

E-mail：jmatsumi@ncc.go.jp

**Research office： Junya Matsumi**

National Cancer Center Hospital

Department of Anesthesia and Intensive Care

E-mail：jmatsumi@ncc.go.jp

2017/07/01 Version 1

2017/08/15 Version 2

2018/01/05 Version 3

2018/02/01 Version 4

2018/05/11 Version 5

2018/05/25 Version 6

2018/07/10 Version 7

2018/09/09 Version 8

2018/10/14 Version 9

2018/10/17 Version 9.1

2018/10/31 Version 9.2

2019/11/07 Version 9.3

2020/04/10 Version 9.4

1. **Summary**
   1. **Schema**

**Metastatic Liver Tumor**

Scheduled hepatectomy in 5 Couinaud segment or less

Between 20 and 90 years old

Preoperative AST・ALT<100IU/L

No simultaneous primary lesion resection, RFA, or biliary reconstruction

**Randamization**

**Group A： Sevoflurane**

**Group B：Propofol**

- 1. **Purpose**

Clarify the effect of intraoperative anesthetics on liver damage during hepatic tumor resection with Pringle maneuver.

- 1. **Patients**

1. Regardless of the primary lesion, metastatic liver tumors (including suspected liver tumors) with number of tumors less than 5 and scheduled resection size with less than 5 Couinaud segments
2. Between 20 and 90 years old
3. Preoperative AST and ALT less than 100 IU/L
4. Primary lesion resection or radiofrequency coagulation is not scheduled at the same time as hepatectomy
5. Not scheduled for biliary reconstruction in addition to hepatectomy
6. Written informed consent has been obtained from the patient to participate in the study
   1. **Intervention**

From preoperative to anesthesia induction and after the end of anesthesia, there are no restrictions in either group. And there are no restrictions other than anesthetics used to maintain anesthesia.

- - 1. **Sevoflurane group**

Use sevoflurane as an anesthetic for the maintenance of anesthesia. Intraoperative dosagee is controlled so that the exhaled sevoflurane concentration is 0.6-2%.

- - 1. **Propofol group**

Use propofol as an anesthetic for the maintenance of anesthesia. Intraoperative dosage is controlled so that the Bispectral index is in the range of 30-70.

- 1. **Endpoint**
     1. **Primary endpoint**

Peak AST measured during 3 days after surgery.

- - 1. **Secondary endpoint**

Peak ALT measured during 3 days after surgery, Postoperative serious adverse event defined by JCOG postoperative complications criteria (Clavien-Dindo Classification) version 2.0 with grade 2 or higher. Peak total bilirubin measured during 3 days after surgery

- 1. **Planned number of registrations and research period**

Planned number of registrations: 56人

Total research period: 4 years

Registration：3 years 3 month, Follow: till 1 month after surgery, Analysis: 8 month

- 1. **Contact**

National Cancer Center Hospital

Department of Anesthesia and Intensive Care

Junya Matsumi

E-mail：jmatsumi@ncc.go.jp

# Purpose

The purpose of this study is to clarify the effects of intraoperative anesthetics on liver tumor resection with Pringle maneuvor.

# 2. Background and rationale for the test plan

## Patient

### Background

In hepatectomy, the Pringle method (a method of temporarily blocking blood flow in the hepatic artery and portal vein system by clamping the hepatic duodenal ligament with forceps or the like) is widely used.^1),2)^ Pringle method reduces intraoperative bleeding, but hepatocytes undergo ischemia-reperfusion injury (IRI). In addition, highly invasive surgery such as hepatectomy causes strong oxidative stress. IRI and oxidative stress cause postoperative liver damage^3)^. General anesthesia is required for hepatectomy, but there are two methods: general anesthesia using volatile anesthetic(VA) and general anesthesia using only intravenous anesthesia (total intravenous anesthesia: TIVA). Volatile anesthetics have been noted to have an ischemic resistance effect. During cardio-plumonary bypass, clinical utility has been recognized not only for reducing myocardial damage but also for reducing postoperative complications. ^4)^ On the other hand, propofol used in TIVA has anti-oxidant effect..^5)^ The results of previous studies in the field of liver surgery are heterogeneous. ^5)-12)^We assess the cause of heterogeneity include interventions for multiple factors affecting IRI(intervention in both ischemia and anesthetics) and heterogeneity in the subjects of study (differences in background liver function, differences in the method of application of the Pringle method).

The golden standard of the marker of liver damage ins transaminases(AST and ALT).^13)^ In many clinical studies, postoperative transaminases are used to evaluate liver damage. ^5)-11)^。However, there are many factors affecting transaminase(for example, the size of the resected liver, the time of hepatic ischemia, liver cirrhosis, fatty liver). Thus, we select the metastatic liver tumor resection with relatively little variation in these factors. And We limit the cases to which the number of tumors less than 5 and scheduled resection size with less than 5 Couinaud segments.

If there are differences in intraoperative anesthetics in this study, selecting the appropriate anesthetic may contribute to reducing liver damage after hepatectomy.

### Current standard general anesthesia methods

There are two anesthesia methods: VA and TIVA. Advantages of VA compared to TIVA are usage of biometric monitoring (end-tidal concentration), less frequency of drug renewal during anesthesia(easy to respond to changes in conditions such as massive bleeding). Advantages of TIVA compared to VA are usage of biometric monitoring (end-tidal concentration), less frequency of postoerative nausea and vomiting). In summary, there is no obvious superiority between VA and TIVA.

## Rationale for setting

### Study treatment of this study

None of the anesthetics used in this study were found to be superior. And the results of previous studies are inconsistent. Thus, both VA and TIVA are standard treatment.

| Author | Journal year | Design | Disease | Group | Result | Limitations |
| --- | --- | --- | --- | --- | --- | --- |
| K. Slankamenac,  et al^10)^ | HPB 2012 | Prospective observational study | HCC | S 141,  P 86 | Peak AST(S629±783 vs. P593±696), ALT, Bilirubin SAE were not significantly different | Continuous ischemia,  Differences in characteristics |
| Beck-Schimne,  et alr^6)^ | Ann Surg 2008 | RCT | HCC | P 34,  P+S(only pre-ischemia) 30 | Peak AST( P733±637 vs P+S508±292 ), SAE were different | Continuous ischemia,  Differences in characteristics |
| Beck-Schimner  et al^7)^ | Ann Surg 2012 | RCT | HCC | P+S (only post-ischemia) with continuous ischemia 48,  P with intermittently ischemia 50,  P with continuous ischemia 17 | Peak AST(P with continuous ishemia 631[386-741] vs. P+S with continuous ischemia443[306-644] vs. P with intermittently ischemia 438[303-549]), SAE were different | Difference in ischemic methods |
| Beck-Schimner,  et al ^11)^ | Transplantation 2015 | RCT | DDLT | P 48, S50  (for recipient) | Peak AST( P925[512-3274] vs. S1097[540-2633]), ALT were not different | No intervention to donor,  Continuous ischemia,、  Difference in ischemic time |
| B. Laviolle,  et al^5)^ | Fundam Clin Pharmac 2012 | RCT | Less than 4 segmentectomy for HCC | P 17,  D 13 | Biomarker of antioxidant was different,  Peak AST and ALT were not significantly different | Difference in ischemic methods |
| Ucar M  et al^12)^ | Transplant Proc 2015 | RCT | LDLT | P 29,  I 24 | Biomarker of antioxidant was different | No outcome data ohter than biomarker for antioxidant |
| JC Song  et al^8)^ | Anesth and Analg 2010 | RCT | HCC | P 50,  S 50 | Peak AST(P581±494 vs. S435±275), ALT, bilirubin, SAE were not different | Continuous ischemia、 |

RCT: randomized controlled study, HCC: hepatocellular carcinoma, DDLT: deceased donor liver transplantation, LDLT: living donor liver transplantation, P: propofol, S: propofol, D:desflurane, I: Isoflurane, SAE:serious adverse event

### Summary of the risk-benefit balance and risk-benefit balance between standard and study treatments

As both VA and TIVA are standard therapy, there is no significant risk-benefit imbalance between VA and TIVA. This study is a superiority test, but it is unclear which drug has superiority.

## Design

### Designing the next planned trial

In the case of this study show that the effect of anesthetics on postoperative liver injury is different, we plan next study with postoperative complications as primary endpoint.

### Rationale for setting up endpoints

The golden standard of the marker of liver damage ins transaminases(AST and ALT).^13)^ In many clinical studies, postoperative transaminases are used to evaluate liver damage. ^5)-11)^ As it was confirmed that the peak values of transaminases were within 3 days after surgery at preliminary retrospective study at our hospital, we set peak transaminases for 3 days after surgery as primary endpoint. As AST seems to be superior than ALT for assessment of liver damage at previous studies, we set AST as primary endpoint and ALT as important secondary endpoint. Referring to previous studies and preliminary study in our hospital(Sevoflurane 658±460 IU/L vs Propofol 351±178 IU/L), the judgement that the degree of liver damage differs depending on the anesthetics was a difference of AST as 300 IU/L..

As both IRI and oxidative stress affect systemically, postoperative adverse event is important outcome. However, as we set metastatic liver tumor resection as target of this study to assess the effect of anesthetics on liver damage, postoperative adverse event may be very low(rate of adverse event as JCOG grade 2 or more after metastatic liver resection is about 1% in our hospital). Thus, we set adverse event as secondary endpoint. And, as massive liver damage may induce prolonged liver dysfunction, postoperative peak total bilirubin is also important endpoint. However, as well as serious postoperative adverse event, the rate of prolonged liver dysfunction after metastatic liver tumor resection is very low. Thus we set peak total bilirubin as secondary endpoint.

### Clinical hypothesis and rationale for setting enrollment

### Clinical hypothesis of this study is “the degree of postoperative liver damage varies depending on the intraoperative anesthetic used in metastatic liver tumor resection with Pringle maneuvor”.

### Postoperative AST is expected to be normally distributed. Clinically, it is judged that a difference of AST 300 IU/L affects the decision of the selection on the anesthetics. Calculate the required number of registrations based on the considerations described later (see “Planned number of registrations, registration period, tracking period”).

### Prospective patient registration

　In our hospital, the number of metastatic liver tumor resection is around 40 per year and about 70% of the patients meet the including criteria. The annual enrollment for this trial is 17 with an expected participation consent acquisition rate of 60%. Thus, the planned registration period is expected to be 3 years and 3 months.

- 1. **Summary of expected benefits and disadvantages of participating in the study**
     1. **Expected benefit**

Patients participating in this study are expected to have no specific benefit.

- - 1. **Anticipated dangers and disadvantages**

The difference between patients participating in this study and usual practice is that they are randomly assigned sevoflulene or propofol as maintenance anesthetics for general anesthesia. However, as regardless of whether or not patients participated in this study, they do not make a decision regarding the choice of anesthesia drug (the anesthesiologist decided), the superiority between VA nad TIVA is not clear and there is no additional testing or treatment due to participation in this study, it is unlikely that the patient will be disadvantaged by participating in this study..

### Significance of this study

### In the case of the difference in postoperative liver injury depending on the intraoperative anesthetic used in this study, selecting an appropriate anesthetic may contribute to the reduction of postoperative liver injury. Moreover, it can be expected to reduce postoperative complications by reducing damage to other organs (lungs, kidneys, etc.).

**3. Criteria and definitions used in this test**

- 1. **Types of hepatectomy**
- major resection：Hepatectomy of 3 or more areas in the Couinaud classification
- minor resection：Hepatectomy of less than 3 areas in the Couinaud classification
  1. **ASA-PS (American Society of Anesthesiologists physical status)**

It is a classification of preoperative general condition defined by the American Society of Anesthesiologists

　Class 1：No complications other than the disease causing surgery

　Class 2：Normal activities of daily living but mild systemic illness

　Class 3：A condition with a high degree of systemic disease that restricts activities of daily living

　Class 4：Conditions requiring life-threatening systemic diseases

　Class 5：A dying condition in which the possibility of saving lives is low even after surgery

　Class 6：Conditions diagnosed as brain dead (brain-dead organ transplant donors)

- 1. **Charlson Index^14)^**

Score to assess for preoperative complications^15)^

- 1. **Postoperative adverse event**

JCOG postoperative complications criteria (Clavien-Dindo Classification) version 2.0 with grade 2 or higher.

# Patient Selection Criteria

Patients who meet all of the following eligibility criteria and do not meet any of the exclusion criteria are eligible for enrollment.

- 1. **Eligibility Criteria**

1. Regardless of the primary lesion, metastatic liver tumors (including suspected liver tumors) with number of tumors less than 5 and scheduled resection size with less than 5 Couinaud segments
2. Between 20 and 90 years old
3. Preoperative AST and ALT less than 100 IU/L
4. Primary lesion resection or radiofrequency coagulation is not scheduled at the same time as hepatectomy
5. Not scheduled for biliary reconstruction in addition to hepatectomy
6. Written informed consent has been obtained from the patient to participate in the study
   1. **Exclusion criteria**
7. There are known contraindications to the drug used

# Enrollment procedure and randomization

- 1. **Enrollment procedure**

Confirm using the registration eligibility confirmation sheet that the target patient meets all the eligibility criteria and does not meet the exclusion criteria, and register at the time of hospitalization for surgical purposes. Register on a password-managed computer of the office in the department of Anesthesia and Intensive Care on the 8th floor of the National Cancer Center Hospital.

Contact:: Research office(Junya Matsumi) TEL：03-3542-2511 FAX：03-3542-2166

- - 1. **Notes on registration**

1. Registration after the start of protocol treatment is not tolerated without exception.
2. Registration is done on a password-managed computer in the Department of Anesthesia and Intensive Care Medicine on the 8th floor of the National Cancer Center Hospital.
3. Eligibility is confirmed using the registration eligibility confirmation sheet, and the confirmation sheet is stored in the Department of Anesthesia and Intensive Care Medicine on the 8th floor of the National Cancer Center Hospital.
4. Registration is completed when two researchers confirm that the contents of the registration eligibility confirmation form and the data at the time of registration are entered on the computer.
5. Except in the case of withdrawal of consent including refusal to use the data for the study, patients once enrolled will not be deregistered.
   1. **Randomization**

Patients will be randomly assigned to enroll by researchers who are not involved in consent and anesthesia. No adjustment factor is provided, and random allocation is performed using the substitution block method.

- 1. **Procedures for Termination of Registration**

When the planned number of registrations is expected to be achieved in this study, the Research Office will inform the researcher..Researchers will not explain to new patients after the planned number of registrations is reached.the Research Office closes registration at 5 p.m. on the Friday following the date on which the expected number of registrations is registered.

# Treatment Plan and Treatment Change Criteria

## To the extent that the patient's safety is not threatened, treatment and modification shall be carried out as described in this chapter. When it is judged to be medically dangerous according to the protocol, the treatment will be changed according to the medical judgment of the attending anesthesiologist. In that case, it is a "protocol deviation", but if it is judged to be medically valid, it is considered to be a "clinically valid deviation".

## Protocol treatment

The drugs used during the maintenance of anesthesia are assigned drugs. The selection and use of other drugs used and anesthesia management, including respiratory circulation management, are performed at the clinical discretion of the anesthesiologist.

- Pringle maneuvor：One course is to block the hepatic artery and portal vein blood flow with the hepatic duodenal ligament for 15 to 30 minutes, and then open it for 5 minutes. And do it as many times as need. For the purpose of reducing IRI, all patients are given intravenous hydrocortisone 100 mg immediately before the initial blockade.
- Induction of anesthesia：Use propofol as a sedative, rocuronium as a muscle relaxant, fentanyl and/or remifentanil as an analgesic.In the case of group A, sevoflurane is started after the patient falls asleep.
- Maintenance of anesthesia：Assign drugs as sedatives, rocuronium as muscle relaxants, fentanyl and/
- or remifentanil and /or epidural anesthesia as analgesics,.
- Awakening of anesthesia：Sugammadex is used for the reverse of muscle relaxants.
- Use of infusion and blood transfusion products, respiratory management such as ventilator setting, and circulation management such as vasopressor use will be performed according to clinical needs, and there will be no restrictions on participation in this study.

### **Sevoflurane group （Group A）**

Propofol is used only during the induction of anesthesia at sevoflurane group. Sevoflurane is controlled at a concentration of 0.6-2% in the exhaled sevoflurane. The use of propofol (except when induction of anesthesia) is prohibited during anesthesia, but there are no restrictions on other drugs.

### **Propofol group （Group B）**

Sedation is performed using propofol from the induction of anesthesia to the end of anesthesia. Propofol is adjusted using BIS monitor 30-70 as a guide. The use of sevoflurane is prohibited during anesthesia, but there are no particular restrictions on other drugs.

## Protocol Treatment Completion and Discontinuation Criteria

## Protocol Definition of Completed Treatment

　　　In this study, the protocol is completed when anesthesia is terminated without departing from the protocol.

## Criteria for Discontinuation of Protocol Treatment

## Discontinue protocol treatment if adverse events are observed in which each group-assigned drug is suspected.

# Expected adverse events

- 1. **Expected adverse events**
     1. **Adverse events expected with the drug**

According to the drug package insert

- - 1. **Adverse reactions and surgical complications expected by surgery**

1. Anesthesia-related complications

Fever^*1^, allergic reaction, inability to secure airway, acidosis^*1^, hyperkalemia^*1^, delirium, hoarseness, hematoma^*2^, catheter-related infection^*3^

*1 Items assuming malignant high fever expected as a complication of general anesthesia

*2 Items assuming epidural hematoma and spinal cord subarachnoid hematoma expected as complications of epidural anesthesia

*3 Items assuming epidural abscess and spinal cord subarachnoid abscess expected as complications of epidural anesthesia

1. Surgical complications

Damage to other organs, biliary fistula (about 10%), postoperative hemorrhage, infection (about 10%), liver failure (less than 0.1%), pulmonary embolism, death (less than 0/1%)

- 1. **Evaluation of adverse events/reactions**

To assess adverse events/reactions, the "JCOG postoperative complications criterion (Clavien-Dindo classification)" is used.

- - 1. **Grading of adverse events**

When grading adverse events, grading is the closest to the definition of grades 0 to 4, respectively. Also, when a specific treatment is described in grade, grading is performed from the clinical necessity. For example, the patient's pleural effusion may be increasing and the patient may refuse oxygen inhalation or chest drainage despite the indication of the situation. In such cases, grading is based on a medical judgment of what should be done, not what was actually done.

# Evaluation items, clinical tests, and evaluation schedule

- 1. **Items to be collected before registration (using information from the anesthesiology preoperative outpatient clinic)**

Patient background (age, sex, primary cancer type, preoperative complications [Charlson Index], presence or absence of preoperative chemotherapy [drug used if performed], allergic history), ASA-PS, biochemical tests (AST, ALT, Bil)

- 1. **Observation period**

The observation period will be up to 28 days after surgery to assess postoperative complications in both groups. If protocol treatment is discontinued in any group, no observation period is set.

- 1. **Items to be collected during the treatment period**

Surgical method, anesthesia method, operative time, anesthesia time, number and total weight of resected liver, total ischemic time, intraoperative blood loss, intraoperative fluid/transfusion volume, intraoperative urine volume.

- 1. **Evaluation items after the end of treatment**
     1. **Primary outcome**
- Liver damage:

Assessed with the highest value of AST 3 days postoperatively.

In addition, the results of postoperative biochemical tests (tested daily for 3 days after surgery) that are usually performed in clinical practice will be used, and blood collection and testing for this study will not be performed.

- - 1. **Secondary outcome**
- Liver damage

As a particularly important secondary endpoint, the highest ALT value at 3 days postoperatively will be evaluated. In addition, the results of postoperative biochemical tests (tested daily for 3 days after surgery) that are usually performed in clinical practice will be used, and blood collection and testing for this study will not be performed.

- Early postoperative liver function evaluation

Assessed at the highest value of Bilirubin 3 days postoperatively. In addition, the results of postoperative biochemical tests (tested daily for 3 days after surgery) that are usually performed in clinical practice will be used, and blood collection and testing for this study will not be performed.

- Postoperative adverse event

JCOG postoperative complication criteria (Clavien-Dindo classification) Collect postoperative complications of grade II or higher for 28 days postoperatively.

- 1. **Evaluation Schedule**

|  | pretreatment | postoperative | | | |
| --- | --- | --- | --- | --- | --- |
|  | Anesthesiology outpatient | Day 1 | Day 2 | Day 3 | 〜 28 days |
| Characteristics | ○ | - | - | - | - |
| ASA-PS | ○ | - | - | - | - |
| AST | ○ | ○ | ○ | ○ | - |
| ALT | ○ | ○ | ○ | ○ | - |
| Bilirubin | ○ | ○ | ○ | ○ | - |
| Postoperative advese event | - | ○ | ○ | ○ | ○ |

1. **Effect Determination and Endpoint Definition**

Including animal experiments, transaminase values are used as an evaluation item for ischemia-reperfusion injury in the liver region.^13)^ In clinical studies on ischemic reperfusion disorder after liver resection, the highest transaminase values in the early postoperative period have been used. ^5)-11)^ Since the purpose of this study is to evaluate ischemic reperfusion damage in the liver region, it will be adopted as a primary endpoint. Based on previous studies including retrospective studies (especially those by Beck-Schimner et al.), AST was considered to be more useful than ALT, and AST was selected as the primary endpoint in this study, and ALT was analyzed as an important secondary endpoint. There is no difference in the effect on postoperative AST and ALT between 15 and 30 minutes for one blood flow blockade.^16)^。

IRI is a condition that can adversely affect the whole body, and serious postoperative complications are considered to be important endpoints. However, since this study included metastatic liver tumor resection to assess the effect of anesthetics on IRI, the incidence of serious postoperative complications is expected to be very low (the incidence of serious complications after metastatic liver tumor resection in our hospital in the last two years is 1%). Therefore, it was set as a secondary endpoint. In addition, Bil, which is also an important endpoint, because severe ischemia-reperfusion injury disorder can cause liver damage from the early postoperative stage. However, as with serious postoperative complications, early postoperative liver damage is very unlikely, so it was set as a secondary endpoint.

- 1. **Define the target population**

Following the intent-to-treat principle, the analysis will be a population consisting of all randomized cases excluding cases with no data on the randomized primary endpoint (Maximum Analysis Set (FAS)).In addition, when discontinuation of protocol treatment occurs, analysis will be performed in a population of patients who have completed protocol treatment (Per Protocol Set: PPS).

- 1. **Defining Endpoints**
- Primary endpoint

The maximum AST value within 3 days after surgery. In a retrospective study of metastatic liver tumor resection in the last 2 years, it was confirmed that the highest value in the early postoperative period was achieved within 3 days after surgery, so it was set as the highest value within 3 days after surgery.

- Secondary endpoint

Set the highest ALT value within 3 days postoperatively as an important secondary endpoint. As other endpoints, the JCOG postoperative complication criterion (Clavien-Dindo classification) Grade II or higher postoperative complications and the highest Bil-value within 3 days after surgery as a liver function evaluation are used.

1. **Reporting Adverse Events and Defects**

In the event of a "serious adverse event" or "unexpected adverse event", the attending physician should report it to the Research Secretariat/Principal Investigator. The events listed in the package insert are not counted as adverse events in this study.

Report to the Minister of Health, Labour and Welfare on adverse reactions based on the Act on Securing Quality, Efficacy and Safety of Pharmaceuticals and Medical Devices (Address: Safety Information Division, Safety Department 1, Pharmaceuticals and Medical Devices Agency, FAX: 0120-395-390, Email: [anzensei-hokoku@pmda.go.jp)](mailto:anzensei-hokoku@pmda.go.jp)17)「,)^[17)](mailto:anzensei-hokoku@pmda.go.jp)17)「,)^[,](mailto:anzensei-hokoku@pmda.go.jp)17)「,) Based on the "Ethical Guidelines for Medical Research Involving Human Subjects" (Ministry of Education, Culture, Sports, Science and Technology / Ministry of Health, Labor and Welfare Notification No. 18), the director of the medical institution reports unexpected serious adverse events to the Minister of Health, Labour and Welfare, etc., and the medical institution to the company to communicate side effects to the company, it is the responsibility of the principal investigator to appropriately report to the head of the medical institution at each facility.

- 1. **Emergency Reportable Adverse Events**

Adverse events that fall under any of the following are designated as "serious".

1. Any death during protocol treatment, regardless of whether or not there is a causal relationship with protocol treatment.
2. Death after completion of protocol treatment and a causal relationship with the protocol cannot be ruled out. Obvious death from primary disease does not apply.
3. Life-threatening:

Grade 4 non-hematologic toxicity or equivalent in CTCAE v4.0.

When hospitalization for treatment or an increase in the duration of admission is required.

However, if hospitalized for the following purposes, it will not be treated as a serious adverse event.

Hospitalization to reduce the burden on patients visiting from remote locations

Pre-planned hospitalization

1. Permanent or severely impaired or dysfunctional.
2. Aplastic anemia. Myelodysplastic syndrome, secondary cancer, or other congenital diseases or abnormalities in later generations
3. Other serious medical events.

Those that do not fall under any of the items 1 to 6 above, but are judged to be medically important by the research responsible physician

- 1. **Reporting Obligations and Reporting Procedures for the Principal Investigator**
     1. **Emergency Report**

When an adverse event that is subject to emergency reporting occurs, the attending physician will promptly notify the Secretariat/Principal Investigator. At that time, care shall be taken not to include the medical record number, etc.

1. Upon learning of an adverse event of CTCAE v4.0-JCOG or Clavien-Dindo Grade 3 or higher, or any other adverse event that is judged to be medically significant, the attending physician should promptly report it to the Secretariat/Principal Investigator.
2. Additional Reporting

When new information is obtained after the above report, the information will be added to the prescribed form and reported as needed.

- - 1. **Report to the head of the medical institution**

When an adverse event that is subject to emergency reporting occurs, the principal investigator reports it to the head of the medical institution as a "serious adverse event" in the "Ethical Guidelines for Medical Research on Human Subjects" in accordance with the regulations of the medical institution.

- - 1. **Reporting to other reporting destinations**

Reporting of product safety information for pharmaceuticals, medical devices, regenerative medicine, etc: Based on Article 68-10, Paragraph 2 of the Act on Securing Quality, Efficacy and Safety of Pharmaceuticals, Medical Devices, etc., information that is deemed necessary to be reported shall be appropriately reported to the Minister of Health, Labour and Welfare in accordance with the regulations of medical institutions.

- 1. **Responsibilities of the Principal Investigator/Secretariat**
     1. **Determining whether registration is suspended and emergency notification is required**

Upon receiving the report from the Principal Investigator, the Research Office will judge the urgency, importance, degree of impact, etc. of the report content, and if necessary, entrust the hospital director and the director to determine whether it is necessary to suspend registration or to notify the facility of information urgently.

- - 1. **Notifying Researchers in the Facility**

The Secretariat/Principal Investigator shall notify the Researcher in the Institute of the decision of the Secretariat/Principal Investigator in writing (e-mail is acceptable) regarding serious adverse events.

1. **Statistics**
   1. **Main analysis and criteria**

The main analysis is to compare the highest AST values within 3 days postoperatively in groups A and B by t-test, and judge a significant difference of less than 0.05 to be statistically significant. As a criterion for determining that the degree of IRI differs depending on the anesthetic, a difference of about AST 300 U / L was observed. This was based on the idea that a difference of about AST300U/L would have a significant impact on clinical anesthetic selection. If necessary, sensitivity analysis will include a population of patients who have completed protocol treatment (PPS), or a population of all randomized cases and supplemented with the worst values observed in groups A and B in cases where primary endpoint data are missing.

- 1. **Planned number of registrations, registration period, tracking period**

Currently, the ischemic method that alleviates IRI is considered to be the intermittent ischemia method, and in consideration of the effect of IRI on the whole body, the intermittent ischemia method is also adopted in our hospital. However, previous studies used continuous ischemia, which strongly expresses IRI, except for one group in the 2012 Beck-Schimner et al. trial, and the mean, median, standard deviation, and quartile values of postoperative AST values are likely to differ from the results of this postoperative ischemia. Therefore, we mainly referred to the results after intermittent ischemia and the results of our retrospective studies in the previous study, and the other studies were only secondary references. First, postoperative AST was treated as normally distributed. Next, clinically, it was judged that a difference of about 300 U/L of AST had a significant impact on anesthetic drug decision. The standard deviation of AST is expected to be 300 U/L. Finally, the significance difference was set to 0.05 and the detection power was set to 0.8. Under these conditions, 28 patients in each group (56 patients in total) were calculated to be required. The number of such surgical cases is about 40 per year at our single facility, and about 70% of them are liver tumor resection in less than 5 places and the liver is completely removed less than 5 areas of Couinaud. The annual enrollment rate for this trial is estimated to be around 17, with an expected participation consent acquisition rate of 60%, and the planned registration period is expected to be 3 years and 3 months. The follow-up period shall be 28 days postoperatively, which is the definition of postoperative complications.

- 1. **Interim analysis and early discontinuation of the study**

　　　　　No early discontinuation of interim analyses or trials is planned.

- 1. **Analysis in secondary endpoints**

As an important secondary endpoint, the highest ALT value within 3 days postoperatively for ischemia-reperfusion injury was compared in two groups by t-test, and a significant difference of less than 0.05 was statistically significant. For postoperative complications within 28 days, the Fisher test compares the two groups, with a significant difference of less than 0.05 as statistically significant. Regarding liver function, the highest bilirubin value within 3 days after surgery was compared in two groups by t-test, and a significant difference of less than 0.05 was considered statistically significant.

- 1. **Final Analysis**

In addition to the analyses described in 10.1 and 10.4, multivariate logistic analyses are performed to exclude the effects of non-anesthesia methods on IRI.

- 1. **End of research**

The research will end with the publication of all papers related to this study.

# Ethical Matters

- 1. **Patient protection**

This research will be conducted in compliance with the Ethical Guidelines for Medical Research on Human Subjects (2016), the Declaration of Helsinki (revised in 2013), and the Act on the Protection of Personal Information (2017).

- 1. **informed consent**
     1. **Explanation to the patient**

Prior to patient registration, the researcher will give the patient an explanatory document approved by the Research Ethics Review Committee and explain the following verbally in detail.

1. About clinical trials and explanatory documents
2. Freedom of Participation

Freedom to refuse consent prior to participation in the research, freedom to withdraw consent once consented, and no unfair medical disadvantages due to these.

1. Medical conditions and treatment of those eligible for this clinical trial

Patients with metastatic liver tumor or suspected hepatectomy are eligible.

1. Significance and purpose of this clinical trial
2. Methods of this clinical trial

To randomly select anesthetics for sustained use in general anesthesia.Except for the difference in anesthetics, it is no different from regular medical care. Collect medical information such as test results and treatment details.

1. About the expected benefits and possible disadvantages

A description of the benefits that you believe you can and the disadvantages you may suffer by participating in this study.

1. The planned duration of the entire clinical trial and the duration of your participation

The participation period must be from the time of surgery to 28 days after the operation.

1. About the expenses you will pay

Explanation of what is similar to general practice：The cost of treatment must be covered by the insurance system.

1. Response and warranty in the event of a health hazard.

Compensation in the event of health damage shall be the same as that of general medical care.

1. Handling of Personal Information

The utmost attention will be paid to the protection of personal information.

1. Publication of Research Results

Publication in academic journals or academic societies in a form that does not identify individuals.Publish the outline, progress, and main results of the research on UMIN. Respond to the disclosure of information from the person to the extent that it does not interfere with the disclosure of information.

1. Funding for this clinical trial and conflicts of interest

This research will be funded by the National Cancer Center Research and Development Fund.The facility and the researchers involved in this research do not have any conflict of interest with companies or organizations related to this research that could damage the credibility of the research.In addition, no specific individual will be able to make any profit from this study.

1. Ethical review of this clinical trial

Approved by the National Cancer Center Research Ethics Review Committee.

1. Research Organization and Contact

The main body of this study is the Department of Anesthesia and Intensive Care, National Cancer Center Hospital, and the person who obtained the explanation and consent will be a doctor belonging to the Department of Anesthesia and Intensive Care.

1. Contact information at our hospital (consultation desk)
   - 1. **Informed consent**

Explain the study and confirm that the patient has a good understanding of the research and then request participation in the study. When the patient agrees to participate in the study, the consent form must be signed by the patient. The researcher confirms that the consent form contains the name of the researcher who gave the explanation and the date of explanation, the name of the patient who received the explanation and consented, and the date of consent.One copy of the consent form will be made and handed over to the patient, and the original will be kept in a safe locked by the principal investigator with a password in the Department of Anesthesia and Intensive Care.

- 1. **Protection of Personal Information and Patient Identification**
     1. **Policies, laws and regulations to be followed by this study**

In conducting this research, we will comply with the following laws and regulations. When laws, norms, or policies other than the following are applicable, they will be followed.

1. Act on the Protection of Personal Information (Act No. 57 of May 30, 2003, last revision: Law No. 65 of September 9, 2015) (1) Act on the protection of personal information (Act No. 57 of May 30, 2003, last revision: Law No. 65 of September 9, 2015)
2. Ethical Guidelines for Medical Research on Human Subjects (Ministry of Education, Culture, Sports, Science and Technology / Ministry of Health, Labor and Welfare Notification No. 3)
3. Declaration of Helsinki (translated by Japan Medical Association)
   - 1. **Purpose of use of personal information, items to be used, and method of use**
4. **Purpose of use**

In this research, we will use the personal information of patients for the purpose of identifying and investigating individual patients in order to obtain correct results of clinical research, and for the purpose of appropriately managing the acquired information.

1. **Items to be used**

The minimum items necessary for patient identification and inquiry (medical record number and medical information) shall be used. No other personal information will be collected, and when it is collected by mistake, it will be destroyed or masked or otherwise processed appropriately to make it illegible.

1. **Methods of use**

Personal information and medical information of patients are collected by researchers by filling in a database. The research registration number linked to the medical record number collected for patient identification and inquiry is used in the database, and the correspondence table is kept by the principal investigator or a person in charge designated by the principal investigator in accordance with the regulations established by the National Cancer Center ("Standard Operating Procedures for Storage of Information on Medical Research in Human Subjects". Also, the database uses a single computer controlled by a password (data is not handled by multiple computers).

- 1. **Data storage**

Medical records and clinic records (electronic medical record data) shall be kept in accordance with the regulations established by the National Cancer Center ("Central Hospital Medical Record Management Regulations"). "Materials pertaining to such information (case reports, records prepared by research subjects, revised records, etc.)" and "Other materials (notification of results of research applications and revisions of research plans by the head of the research institution, implementation status reports, adverse event reports, versions of research plans and explanatory consent documents, etc.)" are in accordance with the regulations established by the National Cancer Center ("Standard Operating Procedures for Supplementing Information in Medical Research in Human Subjects"). The data related to this study will be retained for 10 years from the end of the study, and will be retained for as long as possible after the expiration date. When samples and information related to this research are destroyed after the storage period has elapsed, they will be anonymized and discarded.When a paper on the analysis results of primary or secondary endpoints is published, a PDF of the paper is submitted to the Planning and Management Office, Research Planning Promotion Department, National Cancer Center Hospital.

- - 1. **Secondary Use of Data**

The data obtained in this study may be used for secondary purposes in a form that is not linked to personally identifiable information.

- - 1. **Safety Management and Responsibility System**

We will appoint a person in charge of privacy protection management and a person in charge of privacy protection, and take various safety management measures to minimize the risk of information leakage when using personal information.

- - 1. **Response to Disclosure of Patient Information**

In principle, the person in charge shall be the person in charge when the patient himself / herself requests disclosure of information related to the privacy of this research organization.

- - 1. **Receiving general inquiries and complaints**

General inquiries and complaints can be received by mail, e-mail, or telephone at the following address.

Inquiries：Department of Anesthesia and Intensive Care, National Cancer Center Hospital, Junya Matsumi

〒104-0045 5-1-1 Tsukiji CHuo-ku, Tokyo, E-mail: jmatsumi@ncc.go.jp TEL：03-3542-2511

- 1. **Compliance with the Protocol**

Researchers participating in this study will comply with this protocol as long as it does not compromise the safety and human rights of patients.

- 1. **Approval by the Ethics Review Committee**

Prior to the commencement of this study, the principal investigator will receive approval from the National Cancer Center Research Ethics Review Committee and the president's research permit. In addition, when revising documents such as implementation plans and consent explanatory documents, the approval of the Research Ethics Review Committee must be obtained as to whether or not it is appropriate to continue this research.

- 1. **Report on the progress of clinical research**

The progress of this clinical study and the occurrence of adverse events are reported to the hospital director once a year.

- 1. **Changes to the contents of the protocol**

Changes or revisions to the implementation plan or explanatory documents shall be approved by the Research Ethics Review Committee prior to the effective effect of the changes.

- 1. **Conflicts of Interest (COIs) Related to Research**

Conflicts of interest of this facility and researchers involved in this study are managed by the Secretariat of the Conflict of Interest Committee of the Cancer Research Center. The Institute and the researchers involved in this research do not have any conflict of interest with companies or organizations related to this research that could undermine the credibility of the research. In addition, no specific individual will be monetized by this study.

- 1. **Compensation**

When unexpected health hazards occur due to participation in this study during or after the end of this study, we will take appropriate measures as in the usual medical care. Medical expenses at that time shall be covered by the patient's insurance coverage, and no compensation will be incurred in this study. For health hazards caused by participating in this study, we will provide appropriate treatment according to the medical condition as well as usual medical care as covered by health insurance. At that time, the patient shall bear the co-payment of medical expenses. In addition, we will not provide economic compensation such as condolence money or various allowances.

- 1. **Intellectual Property**

The results, data, and intellectual property rights obtained from this clinical study belong to the National Cancer Center.

- 1. **Disclosure of information about this study**

In principle, the researcher shall respond to requests from the patient himself / herself to disclose information on the privacy of this research organization. When the patient himself wishes, feedback on the results of this research as a whole will be provided and explain that it will be the content of the paper. When the research results are given back and published, the research subjects shall not be identified at all.In addition, the outline, progress, and main results of the study will be published in UMIN-CTR (www.umin.ac.jp/ctr/).

# Monitoring and Auditing

- 1. **Periodic Monitoring**

Annual monitoring will be conducted by an anesthesia and intensive care physician designated by the principal investigator and reported according to the National Cancer Center Hospital Monitoring Report template. In addition, the monitoring staff of the Planning and Management Office, Research Planning Promotion Department, Clinical Research Support Division, National Cancer Center will conduct site monitoring to directly view medical records and various documents two months after the date of approval for research implementation and when half of the scheduled registration period is met.

- 1. **Auditing**

Accept internal audits conducted by the National Cancer Center Research Audit Office.

# Presence of unapproved drugs, unapproved medical devices, off-label drugs, off-label medical devices, and medical practices not permitted by health insurance

It does not include unapproved drugs and medical devices, off-label drugs and off-label medical devices, and medical practices not approved by health insurance.

# Special Notes

None

# Research Organizations

- 1. **Principal Investigator**

National Cancer Center Hospital, Department of Anesthesia and Intensive Care, Junya Matsumi

- 1. **Research Office**

National Cancer Center Hospital, Department of Anesthesia and Intensive Care Junya Matsumi

5-1-1 Tsukiji Chuo-ku, Tokyo TEL: 03-3542-2511 FAX: 03-3542-2116

- 1. **Research Facilities**

National Cancer Center Hospital, Department of Anesthesia and Intensive Care,

- 1. **Funding for this research**

National Cancer Center Resarch Fund (29-A-12 Study on perioperative management and Patient Flow Management optimization of elderly cancer patientsNational Cancer Center Hospital, Department of Anesthesia and Intensive Care, Junya Matsumi, 2017) is a source of funding.

- 1. **プロトコール作成**

National Cancer Center Hospital, Department of Anesthesia and Intensive Care, Junya Matsumi

National Cancer Center Hospital, Department of Anesthesia and Intensive Care, Tetsufumi Sato

National Cancer Center Hospital, Department of Hepatobiliary and Pancreatic, Kazuaki Shimada

National Cancer Center Hospital, Department of Hepatobiliary and Pancreatic, Minoru Esaki

National Cancer Center Hospital, Department of Hepatobiliary and Pancreatic, Satoshi Nara

National Cancer Center Hospital, Department of Hepatobiliary and Pancreatic, Youji Kishi

- 1. **Researcher**

National Cancer Center Hospital, Department of Anesthesia and Intensive Care, Junya Matsumi

National Cancer Center Hospital, Department of Anesthesia and Intensive Care, Tetsufumi Sato

National Cancer Center Hospital, Department of Anesthesia and Intensive Care, Junya Matsumi

National Cancer Center Hospital, Department of Anesthesia and Intensive Care, Tetsufumi Sato

National Cancer Center Hospital, Department of Hepatobiliary and Pancreatic, Kazuaki Shimada

National Cancer Center Hospital, Department of Hepatobiliary and Pancreatic, Minoru Esaki

National Cancer Center Hospital, Department of Hepatobiliary and Pancreatic, Satoshi Nara

National Cancer Center Hospital, Department of Hepatobiliary and Pancreatic, Takeshi Takamoto

# Presentation of research results

After the publication of all papers related to this study, the results will be promptly reported to the head of the research institution. The results of this study will be presented in papers at academic societies in Japan and abroad and in medical journals with referees. In principle, the author of the main published paper of the research result shall be the principal investigator, and the group representative shall be determined according to the degree of contribution in accordance with the restrictions imposed by the submission regulations of the paper, and the final author shall be Tetsufumi Sato.

# References

1. Pringle JHV. Notes on the arrest of hepatic hemorrhage due to trauma. Ann Surg. 1908: 48; 541-549
2. van der Bilt JD, et al. European survey on the application of vascular clamping in liver surgery. Dig Surg. 2007; 24: 423-435.
3. Clavien PA, et al. Strategies for safer liver surgery and partial liver transplantation. N Engl J Med. 2007; 356: 1545-1559
4. Uhlig C, et al. Effects of volatile anesthetics on mortality and postoperative pulmonary and other complications in patients undergoing surgery. Anesthesiology 2016; 124: 11230-1245.
5. Laviolle B, et al. Effect of an anesthesia with propofol compared with desflurane on free radical production and liver function after partial hepatectomy. Fundam Clin Phrmacol 2012; 26: 735-742.
6. Beck-Schimmer B, et al. A randomized controlled trial on pharmacological preconditioning in liver surgery using a volatile anesthetic. Ann Surg 2008; 248: 909-918.
7. Beck-Schimmer B, et al. Protection of pharmacological postconditioning in liver surgery. Ann Surg 2012; 256: 837-845.
8. Song JC, et al. A comparison of liver function after hepatectomy with inflow occlusion between sevoflurane and propofol anesthesia. Anesth Analg 2010; 111: 1036-1041.
9. Ko JS, et al. The effects of desflurane and propofol-remifentanil on postoperative hepatic and renal functions after right hepatectomy in liver donors. Liver transpl 2008; 14: 1150-1158.
10. Slankamenac K, et al. Does pharmacological conditioning with the volatile anaesthetic sevoflurane offer protection in liver surgery? HPB 2012; 14: 854-862.
11. Beck-Schimmer B, et al. Conditioning With Sevoflurane in Liver Transplantation: Results of a Multicenter Randomized Controlled Trial. Transplantation 2015; 99: 1606-1602.
12. Ucar M, et al. Comparison of antioxidant effects of isoflurane and propofol in patients undergoing donor hepatectomy. Transplant Proc 2015; 47: 469-472
13. Iu S, et al. Markers of allograft viability in the rat: relationship between transplantation viability and liver function in the isolated perfused rat liver. Transplantation 1987; 45: 562-569.
14. Charlson ME, et al. A new method of classifying prognostic comorbidity in longitudinal studies: development and validation. J Chronic Dis. 1987; 40: 373-383.
15. Khandoga A, et al. Differential significance of early surgical complications for acute and long-term recurrence-free survival following surgical resection of hepatocellular carcinoma: do comorbidities play a role? Eur J Gastroenterol Hepatol. 2017; 29: 1045-1053.
16. Esaki M, et al. Randomized clinical trial of hepatectomy using intermittent pedicle occlusion with ischaemic intervals of 15 versus 30 minutes. Br J Surg. 2006; 93: 944-951.
17. “Report to the Minister of Health, Labour and Welfare on adverse reactions based on the Act on Securing Quality, Efficacy and Safety of Pharmaceuticals and Medical Devices" http://www.info.pmda.go.jp/info/houkoku.html
18. "Ethical Guidelines for Medical Research on Human Subjects" http://www.mhlw.go.jp/stf/seisakunitsuite/bunya/hokabunya/kenkyujigyou/i-kenkyu/index.html
