# Supplementary material for: Protective effect of propofol compared with sevoflurane on liver function after hepatectomy with Pringle maneuver: A randomized clinical trial
Source: PLoS One. 2023 Aug 24;18(8):e0290327. doi: 10.1371/journal.pone.0290327 (PMC10449203; doi:10.1371/journal.pone.0290327)
Supplement: Attachment — Submitted filename: renamed_2a3c7.docx [file pone.0290327.s009.docx]

# COMMENTS – Manuscript PONE-D-23-10418

Title: “Protective effect of propofol compared with sevoflurane on liver damage during liver resection with Pringle maneuver: a randomized clinical trial”

**Important note: This review pertains only to ‘statistical aspects’ of the study and so ‘clinical aspects’ [like medical importance, relevance of the study, ‘clinical significance and implication(s)’ of the whole study, etc.] are to be evaluated [should be assessed] separately/independently. Further please note that any ‘statistical review’ is generally done under the assumption that (such) study specific methodological [as well as execution] issues are perfectly taken care of by the investigator(s). This review is not an exception to that and so does not cover clinical aspects {however, seldom comments are made only if those issues are intimately / scientifically related & intermingle with ‘statistical aspects’ of the study}. Agreed that ‘statistical methods’ are used as just tools here, however, they are vital part of methodology [and so should be given due importance]. I look at the manuscript in/with statistical view point, other reviewer(s) look(s) at it with different angle so that in totality the review is very comprehensive. However, there should be efforts from authors side to improve (may be by taking clues from reviewer’s comments). Therefore, please do not limit the revision only (with respect) to comments made here.**

**COMMENTS:** Although it is observed that this manuscript is well drafted [and the study is excellent with respect to most of the aspects], I have few observations/concerns (different opinion) which are given below:

I have few (minor/little) doubts regarding ‘Sample size estimation’. Firstly, you said “We set a difference of 300 IU/L in postoperative peak AST levels between the two groups” but the basis of this is not given. Though later as you specified since it is felt to be ‘clinically significant’ is perfectly alright but mind you that unless any reference is quoted, it is treated as ‘subjective’ [I am not a clinician, however, general opinion is expressed]. For standard deviation you said “was decided by referring to retrospective data of 52 patients” but the source of these data is not revealed. At the end of this section you said “this study was adequately powered with 28 patients per group (total 56 patients)” is doubtful because according to table-2 on page 158 of Jacob Cohen’s paper “A power primer” in Psychological Bulletin, 1992, vol.:112, pp 155-159 [which is a sort of summary of the excellent book by Cohen himself titled ‘Statistical power analysis for the behavioral sciences’, Academic Press, 1977, New York] even for medium effect size you need n=64 per group (type-I error=0.05, power=80%). Fortunately, the power achieved in this trial is not very low (75.69% by referring to table-2 of this manuscript), however, here the comparison pertains only to ‘post’ values and baseline is not given any recognition [I am not sure regarding its clinical importance/relevance]. Look at the absolute values [both mead & S.D.] in table-2 {Peak AST level (IU/l)→ Sevoflurane group (n=28) 510.3 [362.4] & Propofol group (n=28) }291.7 [151.2]}.

Any software or manual calculations will show that your ‘sample size’ results are definitely correct [as an example, a ‘Screen-shot’ of output from software called COMPARE2 is pasted below – which shows that I have no doubt about correctness of calculations], I doubt only about assumptions [assumed values of a difference of 300 IU/L and a standard deviation of 400 IU/L]. This implies that a very large effect size is assumed and that is doubtful.

Example: Screen-shot of output from COMPARE2

Note that the comparison of baseline characteristics when random allocation/assignment is used/done is not required [‘P’ values in last column 4 of Table 1 - Patients characteristics]. In this context, please read [though I am sure that the authors already know these things] a note which is pasted from one famous standard textbook on ‘Medical Research Methodology’:

To provide a description of baseline characteristics is entirely reasonable (since it is clearly important in assessing to whom the results of the trial can be applied), however, statistical comparison of baseline characteristics when random allocation/assignment is used/done [often for good/standard/leading journals these days] is not required, because even if P-value(s) turn(s) out to be significant (while comparing baseline characteristics despite random allocation), it is, by definition, a false positive as you then are supposed to be testing ‘randomization’ then, which in any single trial may not balance all baseline characteristics (particularly when sample sizes are small). Remember that ‘randomization’ is a sort of ‘insurance’ and not a guarantee scheme. Authors may please refer to following articles:

References:

1. Stuart J. Pocock, et al., ‘Subgroup analysis, covariate adjustment and baseline comparisons in clinical trial reporting: current practice and problems’, Statistics in medicine, 2002; 21:2917–2930 [Particularly page 2927]
2. Harrington D, et al., ‘New guidelines for statistical reporting in the journal’, N Engl J Med 2019;381:285-6

[Important message (indirectly/ultimately indicated) from these articles: Never do any comparison with respect to ‘baseline’ characteristics {by applying statistical significance test(s)}, when allocation is done randomly].

However, Statistical comparison [only with respect to important/indicated variables] of baseline characteristics may be performed, to find out if analysis adjustment (say stratified analyses or else) is required with respect to these variables.

In ‘Abstract- Results’ section [and also in ‘outcome - Per-protocol analysis’ section later] there is a sentence “At multiple linear regression …..” which, in my opinion, is not grammatically correct {instead of At, it should be As shown by} . Please check. Later in ‘Randomization’ section, a sentence “A computer generated the blocked randomization with no stratification” appears to be incomplete. In section ‘Statistical analyses’, instead of “We expressed the assessed variables….” it could/should [desired] be “We summarised the assessed variables….”, I think.

Highlighting (listing/pointing out) the “Limitations” {with possible effects} is highly appreciable (although lower sample size is not included & starting ‘Discussion’ with “In this adequately powered RCT is not agreed). However, mind you that as pointed out in ‘important note’ above “This review pertains only to ‘statistical aspects’ of the study and so ‘clinical aspects’ should be assessed separately/independently [preferably by an expert surgeon in concerned field]. Some of these limitations may have significant influence on results.

Except these minor points, the article is acceptable. ‘Minor Revision’ is recommended.
